# Single-cell transcriptomics reveals immune remodeling of the murine lung microenvironment following chronic house dust mite exposure

**DOI:** 10.3389/fimmu.2025.1652539

**Published:** 2025-11-27

**Authors:** Han Chang, Liping Zeng, Zahra Malakoutikhah, Chanond A. Nasamran, Scott Herdman, Maripat Corr, Kathleen M. Fisch, Nicholas J. G Webster, Eyal Raz, Samuel Bertin

**Affiliations:** 1 Division of Rheumatology, Autoimmunity, and Inflammation, Department of Medicine, University of California, San Diego, La Jolla, CA, United States; 2 Center for Computational Biology and Bioinformatics, School of Medicine, University of California, San Diego, La Jolla, CA, United States; 3 Division of Endocrinology, Department of Medicine, University of California, San Diego, La Jolla, CA, United States; 4 Medical Research Service, Veteran Affairs San Diego Healthcare System, San Diego, CA, United States; 5 Moores Cancer Center, University of California, San Diego, La Jolla, CA, United States

**Keywords:** single-cell RNA sequencing, house dust mite, interleukin-1β, chronic inflammation, lung immune microenvironment

## Abstract

**Introduction:**

House dust mite (HDM) is a common environmental aeroallergen strongly associated with asthma and chronic airway inflammation. While HDM exposure is known to induce T helper 2 (Th2)-mediated eosinophilic inflammation, its chronic effects on interleukin-1β (IL-1β)-associated neutrophilic inflammation remain poorly understood. This study used single-cell RNA sequencing (scRNA-seq) to investigate how chronic HDM exposure remodels the murine lung immune microenvironment, with a focus on the role of IL-1β in shaping HDM-driven immune responses.

**Methods:**

Wild-type and *Il1b*-deficient mice on a C57BL/6 background were subjected to chronic HDM exposure over a 5-week period, and scRNA-seq was performed to characterize changes in the lung immune landscape. To enhance resolution of specific cell populations and better define their functional states, we performed subclustering, followed by pathway enrichment and transcription factor activity analyses. To account for endotoxin-dependent variability in HDM extracts, scRNA-seq was conducted using a low-lipopolysaccharide (LPS) HDM extract, while validation studies (histopathology, RT-qPCR, ELISA, and immunofluorescence) were performed using a high-LPS HDM extract.

**Results:**

Our analysis demonstrates that chronic HDM exposure promotes the recruitment and activation of diverse immune cell populations in the lungs, including neutrophils, M2-polarized macrophages, B-2 (follicular) B cells, and multiple subsets of regulatory and effector CD4⁺ T cells. These populations contribute differently to the development or resolution of chronic lung inflammation through IL-1β-dependent and -independent mechanisms. scRNA-seq indicated that IL-1β signaling is critical for sustaining neutrophil and Th17 responses, whereas *Il1b* deficiency promotes Th2-skewed polarization. Validation experiments revealed that these effects are influenced by the endotoxin content of HDM extracts, which can override genotype-dependent effects.

**Discussion:**

These findings demonstrate that chronic HDM exposure profoundly remodels the lung immune microenvironment through IL-1β-dependent and -independent mechanisms. The effects are context-dependent and modulated by the endotoxin content of HDM extracts, highlighting the complex immunomodulatory effects of HDM in inducing chronic lung inflammation.

## 1 Introduction

Exposure to house dust mites (HDM), among the most prevalent indoor aeroallergen worldwide, induces allergic reactions and lung inflammation through both innate and adaptive immune mechanisms (1). Allergens from HDM species such as *Dermatophagoides pteronyssinus* are implicated in 50-85% of asthma cases (1–3). HDM extracts contain various bioactive components, including proteolytic enzymes from the mites’ digestive system, lipopolysaccharide (LPS) from Gram-negative bacteria, and chitin derived from mite exoskeletons and fecal pellets (1). While many studies have examined the effects of acute HDM exposure in mice using short-term asthma models (4), fewer have explored the immune consequences of chronic exposure. Repeated intranasal (i.n.) HDM instillations recapitulates key features of human asthma, including airway obstruction, hyperresponsiveness, eosinophilic and CD4^+^ T helper 2 (Th2) cell infiltration, mucus hypersecretion, and airway remodeling (1, 4). For example, i.n. HDM instillation five days per week for seven weeks induces persistent inflammation, characterized by eosinophil, dendritic cell, macrophage, and Th2 cell infiltration, along with epithelial metaplasia, smooth muscle hyperplasia, and fibrosis (5, 6). HDM-specific Th2 cells promote allergic responses by secreting IL-4, IL-5, and IL-13, which drive IgE production and eosinophilic inflammation (1). Interestingly, long-term HDM exposure (11 weeks) can suppress airway eosinophilia and hyperreactivity while increasing Foxp3^+^ regulatory T cells and IL-10^+^ alveolar macrophages, indicating a shift toward immune regulation (7). HDM also stimulates IL-1β production by monocytes, which drives lung epithelial secretion of pro-inflammatory mediators such as GM-CSF, IL-6, IL-25, IL-33, and TSLP, further amplifying lung inflammation (1, 8). Beyond asthma, IL-1β contributes to COPD, lung fibrosis, and lung cancer (9–11), suggesting that chronic HDM exposure may promote other inflammation-driven lung diseases through this axis.

While the positive association between asthma and lung cancer remains controversial (12, 13), and HDM exposure has not been causally linked to lung cancer development in humans, we recently identified that chronic i.n. instillation of HDM extracts accelerates lung cancer development and progression in the two different mouse models of lung cancer (14). Mechanistically, the lung cancer-promoting effect of HDM was primarily attributed to the chronic activation of the Nod-like receptor family pyrin domain-containing protein 3 (NLRP3) inflammasome in lung macrophages, leading to persistent IL-1β production in the lungs. Notably, pharmacological blockade or genetic deletion of NLRP3, caspase-1, IL-1β, or C-C motif chemokine ligand 2 (CCL2), which contribute to the recruitment of circulating bone marrow-derived monocytes and to the replenishment and pro-inflammatory phenotype of lung macrophages, almost completely abrogated the lung cancer-promoting effect of HDM in our models. Based on these findings, we hypothesize that chronic exposure to HDM and subsequent activation of the IL-1β signaling pathway change lung microenvironment and make it conducive to tumor growth.

The lung immune microenvironment is a complex niche composed of diverse immune cell populations, which makes it challenging to elucidate the function of individual cell types. However, recent advances in single-cell RNA sequencing (scRNA-seq) technology and bioinformatics have made it possible to identify various immune cell types and subtypes and to gain insight into their specific functions within the lung microenvironment (15, 16). In this study, we employed scRNA-seq to investigate how HDM exposure and IL-1β signaling reshape the lung immune microenvironment. Our analysis identified diverse immune cell populations that respond differentially to HDM exposure through IL-1β-dependent or -independent mechanisms. To validate these findings, we also performed complementary histopathology analysis, RT-qPCR, ELISA, and immunofluorescence staining. Together, these data provide a detailed view of the cellular and molecular responses following chronic HDM exposure in the murine lung and reveal context-dependent roles of IL-1β signaling in modulating the lung immune landscape.

## 2 Materials and methods

### 2.1 Mice

Wild-type (WT) C57BL/6J (JAX, Strain# 000664, RRID: IMSR_JAX:000664) were initially purchased from the Jackson laboratory (JAX). Initial breeding pairs of *Il1b^–/–^* (JAX, Strain# 034447, RRID: IMSR_JAX:034447) backcrossed to C57BL/6 for over 10 generations were a gift from Dr. Wai Wilson, Dr. Cheung Robert, and Dr. Hal Hoffman (all from UC San Diego). The mice were bred in our vivarium under specific pathogen-free (SPF) for more than 6 months and were genotyped before they were used in any experiments. All mice were kept on a 12-hour light and 12-hour dark cycle with a standard chow diet and water. All the animal studies were conducted in accordance with protocols approved by the Institutional Animal Care and Use Committee (IACUC) at the University of California San Diego (Protocol Number: S02240) and adhered to the Animal Research: Reporting of *In Vivo* Experiments (ARRIVE) guidelines.

### 2.2 Allergen extract solubilization

Lyophilized extracts of HDM (*Dermatophagoides pteronyssinus*) (Greer laboratories, Cat# XPB82D3A25) were resuspended based on their total protein content at the concentration of 2 mg/mL in sterile 0.9% sodium chloride solution (BD, Cat# 306546). The extracts were then aliquoted and stored at -80 °C until used. The concentrations of total protein, Der p1 and LPS in the HDM extracts are summarized in **Supplementary Table 1**.

### 2.3 Chronic HDM exposure model

Three-to-five months old male C57BL/6 WT or *Il1b^–/–^* mice were first sensitized i.n. under light anesthesia (isoflurane) with HDM (50 μg/50 μL/mouse) or the control vehicle (VEH; 0.9% sodium chloride solution) (50 μL/mouse) on day 0 and were then challenged i.n. 3x/week for the subsequent 4 weeks with HDM (12.5 μg/50 μL/mouse) or with VEH (50 μL/mouse) as shown in **Supplementary Figure 1**. The mice were euthanized by CO2 asphyxiation 24 hours after the last i.n. challenge and the lungs were harvested. In one experiment, the whole lung (all five lobes) was used for scRNA-seq analysis. In a separate experiment, following ligation, one lung lobe of the right lung was removed, snap-frozen in liquid nitrogen, and stored at - 80 °C until processing for RNA and total protein extraction. The remaining four lung lobes were perfused via cardiac perfusion with 5 mL HBSS containing 1 mM EDTA, fixed by intratracheal instillation and immersion in 10% buffered formalin for 24h, and then transferred to histological-grade 70% ethanol until paraffin embedding.

### 2.4 Histological analysis

The fixed lung samples were brought to the UC San Diego Moores Cancer Center Tissue Technology Shared Resource for paraffin embedding and sectioning. The fixed lungs were cut into 4-6-μm sections, placed on glass slides, and stained with hematoxylin (Thermo Fisher Scientific, Cat# 7221) and eosin (Thermo Fisher Scientific, Cat# 7111) (H&E) on a Gemini AS slide stainer (Thermo Fisher Scientific) using standard staining procedures. H&E-stained slides were scanned on an Aperio AT2 slide scanner (Leica Biosystems) to generate whole-slide digital images. Images were acquired and analyzed using QuPath (v0.5.1**;** RRID: SCR_018257). Histopathological analysis and inflammation scoring were performed in a blinded fashion by a board-certified pathologist. The degree of inflammation was assessed using a semiquantitative scale (14). Briefly, the extent of inflammatory cell infiltrates was graded from 0 to 4 as follows: 0 = absent, 1 = very little (<10% of the lung surface area), 2 = mild (10-25%), 3 = moderate (25-50%), and 4 = severe (>50%).

### 2.5 Reverse transcription quantitative PCR analysis

Isolation of total RNA from lung tissues was carried out with the PureLink RNA Mini Kit (Thermo Fisher Scientific, Cat# 12183018A) following the manufacturer’s protocol. One μg of RNA sample was used for reverse transcription and cDNA synthesis using qScript cDNA SuperMix (Quanta Biosciences, Cat# 95048). qPCR was performed on a QuantStudio 3 Real-Time PCR System (Thermo Fisher Scientific, Cat# A28137) using PowerUp SYBR Green Master Mix (Thermo Fisher Scientific, Cat# A25742) according to the manufacturer’s instructions. Samples were normalized to *Rplp0* (36B4) gene expression as indicated in the figure legends. qPCR primers for specific target genes were designed based on their reported sequences and synthesized by Integrated DNA Technologies (IDT) Technologies. See **Supplementary Table 2** for a list of the oligonucleotide sequences.

### 2.6 Enzyme-linked immunosorbent assay

To evaluate cytokine concentration in lung tissue homogenates, ~15–30 mg of lung tissue was minced into small pieces and homogenized for 1x or 2x 15 sec in ~200-400 µL of RIPA buffer (Thermo Fisher Scientific, Cat# 89901) supplemented with protease inhibitors (Roche, Cat# 11836170001). The samples were kept on ice for 30 min and centrifuged at 12,000 rpm for 15 min. The pellets were discarded, and the total protein concentration was determined in the supernatants with a protein quantification kit (Pierce BCA Protein Assay Kit, Cat# 23250). Two hundred μg of total proteins diluted into 50 μL of 1X ELISA diluent (Thermo Fisher Scientific, Cat# 00-4202) was used to determine cytokine levels using target-specific ELISA kits for IL-1β (R&D Systems, Cat# DY401-05), IL-4 (Thermo Fisher Scientific, Cat# 88-7044-88), IL-13 (Thermo Fisher Scientific, Cat# 88-7137-88), IL-17A (Thermo Fisher Scientific, Cat#88-7371-88), and TNF (Thermo Fisher Scientific, Cat# 88-7324-88) following the manufacturer’s instructions.

### 2.7 Immunofluorescence staining

Formalin-fixed paraffin embedded (FFPE) tissue sections were deparaffinized in Xylene. Antigen retrieval was performed in citrate buffer (pH 6.0) in a 96°C water bath for 10 min, followed by permeabilization with 0.2% Triton X-100 in PBS for 5 min. For NETs staining, slides were blocked in TBS containing 5% goat serum and 1% BSA and stained overnight at 4°C with mouse anti-mouse neutrophil elastase (Santa Cruz Biotechnology, Cat# sc-55549) and rabbit anti-mouse histone H3 (cirtrulline R17+ R2 +R8) (Abcam, Cat# AB281584) antibodies in TBS with 0.05% Tween-20 and 1% BSA. After washing, slides were incubated for 1h at room temperature in the dark with goat anti-mouse AF488 antibody (Abcam, Cat# AB150113) and goat anti-rabbit AF647 (Thermo Fisher Scientific, Cat# A21244) secondary antibodies. For Th2 and Th17 cell staining, slides were blocked in PBS containing 5% goat serum and 1% BSA and stained overnight at 4°C with mouse anti-mouse CD3ε antibody (Santa Cruz Biotechnology, Cat# sc-20047), rat anti-mouse IL-4 (BioXcell, Cat# BE0045), and rabbit anti-mouse IL-17A antibody (Thermo Fisher Scientific, Cat# PA5-114455) in PBS with 0.2% Triton X-100 and 1% BSA. After washing, slides were incubated in the dark for 1h at room temperature with goat anti-mouse AF488 (Abcam, Cat# AB150113), goat anti-rat AF647 antibody (Thermo Fisher Scientific, Cat# A-21247), and goat anti-rabbit AF568 (Abcam, Cat# AB175471) secondary antibodies. A negative control was included by staining one tissue section with the secondary antibodies alone. Slides were counterstained with DAPI (50 ng/mL, Invitrogen) for 10 min at room temperature in the dark, rinsed with deionized water, and mounted using ProLong Gold antifade reagent (Thermo Fisher Scientific, Cat# P36930). Slides were scanned using an Olympus VS200 slide scanner (Olympus Life Science). Image analysis and quantification were performed using QuPath (v0.5.1; RRID: SCR_018257). Neutrophils (neutrophil elastase [NE]-positive cells), neutrophil extracellular traps (NETs; NE and citrullinated histone H3 double-positive cells and filamentous structures), Th2 (CD3 and IL-4 double-positive cells), and Th17 (CD3 and IL-17A double-positive cells) cells were quantified in a blinded manner using QuPath’s positive cell detection tool. The average number of positive cells across four lung lobes per mouse was calculated and expressed as the number of positive cells per total lung area (mm²). n = 3–4 mice per group were analyzed.

### 2.8 Single-cell suspensions and fluorescence-activated cell sorting

Lung single-cell suspensions were prepared as previously described (14). Briefly, mice lungs were perfused via cardiac perfusion with 5 mL HBSS containing 1 mM EDTA, harvested, minced using scissors, and enzymatically digested at 37 °C for 30 min in HBSS containing 0.5 mg/mL collagenase type IA (Sigma, Cat# C9891), 20 μg/mL DNase I Type IV (Sigma, Cat# D5025), 5% FCS, 100 U/mL penicillin, and 100 μg/mL streptomycin (Sigma, Cat# P4333). After RBC lysis using ACK buffer for 1 min on ice, the cells were passed through a 100 μm strainer and the single-cell suspensions from n = 3–4 mice/group were pooled together. Cells were counted and resuspended in PBS/2% FCS, then stained with Annexin V-PE (BioLegend, Cat# 640908) and DAPI (Thermo Fisher Scientific, Cat# 62248) following the manufacturer’s instructions. Cell sorting was performed on a BD FACS Aria II cell sorter (BD Biosciences, RRID: SCR_018934) to eliminate transcriptional noise from dead and dying cells. Live single cells, defined as Annexin V/DAPI double-negative cells, were sorted with >98% purity and accounted for approximately 20% of total singlet populations. The cells were counted using trypan blue exclusion and resuspended at a concentration of 1000 cells/μL in PBS/0.04% BSA (Thermo Fisher Scientific, Cat# AM2616). The cell suspension was transferred to 1.5 mL RNA/DNA LoBind Eppendorf tubes (Thermo Fisher Scientific, Cat# 13-698-791) and processed immediately for scRNA-seq.

### 2.9 Single-cell library preparation and sequencing

A total of 16,500 cells per sample were loaded in duplicate onto the Chromium Controller (10x Genomics) using the Single Cell 3’ v3.1 Dual Index kit (Cat# 1000268), targeting a recovery of ~10,000 cells per sample. Four biological conditions were each processed in duplicate on a single 10x chip for Gel Beads-in-Emulsion (GEM) generation. Barcoding, GEM-RT cleanup, cDNA amplification, and library preparation were carried out according to the manufacturer’s protocol at the UC San Diego IGM Genomics Center (RRID: SCR_022740). Post-library quality control was performed using Agilent High Sensitivity D1000 ScreenTape. Sequencing was conducted on the Illumina NovaSeq 6000 S4 platform. All samples achieved high-quality metrics, including Q30 scores >90% from Illumina sequencing, and sequencing saturation >80% and mean mapping rate of 94.9% to the *Mus musculus* reference genome (mm10-2020-A) as reported by Cell Ranger v7.0.1 (10x Genomics, RRID: SCR_017344).

### 2.10 Single-cell data preprocessing

Cell Ranger v7.0.1 (10x Genomics, RRID: SCR_017344) was used for demultiplexing, alignment, and unique molecular identifier (UMI) quantification. Of the 65,024 cells captured, an average of 218 million reads per sample and a median of 2,524 UMIs per cell were obtained. Preprocessing and analysis were conducted using the Seurat R package (17). SoupX was first applied to CellRanger objects to correct for ambient RNA during droplet-based capture process (18). Cells were then excluded with Seurat if they exhibited >5% mitochondrial gene content, <300 or >2,500 detected genes, or <600 UMIs (or above the mean + 2 SDs). DoubletFinder was lastly conducted to remove heterotypic doublets (19). After quality control and filtering, a total of 51,139 high-confidence cells were retained for downstream analysis; the raw read counts, specific quality control metrices, and filtered cell counts per sample can be found in **Supplementary Table 3**.

### 2.11 Clustering and identification of cell types

Each dataset was independently normalized using “SCTransform” and integrated using “FindIntegrationAnchors” and “IntegrateData” functions implemented in Seurat R Package (20, 21). Dimensionality reduction and clustering were performed using Uniform Manifold Approximation and Projection (UMAP) with a resolution of 0.5, yielding initial 17 clusters. Top differentially expressed genes (DEGs) for each cluster were identified using the “FindAllMarkers” function in Seurat, based on Wilcoxon Rank Sum Test, and visualized in heatmaps highlighting the top five DEGs per cluster. To identify conserved cell type markers across conditions, we used the “FindConservedMarkers” function in Seurat. Cell type annotations were assigned based on cluster-specific DEGs, conserved gene expression profiles, and canonical marker genes reported in murine lung scRNA-seq studies. These annotations were further validated using external reference resources including the SingleR package, GeneCards, and the ImmGen database (22, 23). To enhance resolution, subclustering was performed on clusters representing similar cell types. Mononuclear phagocytes and stromal cells were subclustered together using UMAP to refine annotations and distinguish closely related cell populations. Dot plots included the canonical and key cell subtype marker genes that were not represented in the heatmaps. For each cell cluster and subcluster, relative cell numbers are shown in the main figures, while absolute numbers are provided in the **Supplementary Figures**.

### 2.12 Differential gene expression analysis

The **“**FindAllMarkers” function in Seurat was used to identify DEGs between clusters across two conditions, using the Wilcoxon Rank Sum test with Bonferroni correction for multiple comparisons. DEGs were defined as genes with an adjusted p-value <0.05 and an absolute log_2_ fold change >0.25. To functionally interpret DEGs within each subcluster, we performed Gene Ontology (GO) enrichment using the “enrichGO” function from the clusterProfiler R package (v4.14.6) with annotations from the org.Mm.eg.db mouse database (24). GO enrichment focused on Biological Process (BP) terms and applied Benjamini-Hochberg correction to control for multiple testing, with significance thresholds set at p <0.05 and q <0.2. Kyoto Encyclopedia of Genes and Genomes (KEGG) pathway enrichment was conducted using enrichKEGG, mapping significant DEGs to murine KEGG terms (25). We employed the decoupleR R Package (v2.3.0) (26) to infer transcription factor (TF) with the DoRothEA v2.0 regulon database (27) and pathway activities with the PROGENy (28). For TF activity inference, we applied the Virtual Inference of Protein-Activity by Enriched Regulon analysis (VIPER) algorithms (29) to compute enrichment scores. For pathway activity inference, we used multivariate linear model approach, integrating PROGENy pathway weights for estimation. Differential activity and False Discovery Rate (FDR)-adjusted p-values were obtained by applying Wilcoxon rank-sum tests followed by Benjamini-Hochberg correction for multiple testing. The top 10 differentially active TFs and PROGENy pathways were visualized using ggplot2 (30).

### 2.13 Data visualization

UMAPs and dot plots were visualized using Seurat (17). Bar plots were plotted by ggplot2 (30). Differential gene expression was visualized using EnhancedVolcano (v1.2.0) (31). Enrichment results from GO and KEGG were visualized via clusterProfiler (24) and enrichplot (v1.26.6) (32). All visualizations were exported as high-resolution figures using the “ggsave()” function in R (ggplot2 package, RRID: SCR_014601) or R base graphics devices.

### 2.14 Statistical analysis

The Wilcoxon rank-sum test was used as a nonparametric method to assess differential gene expression, GO terms, pathway enrichment, and TF activity. p-values were adjusted by the Bonferroni correction, and adjusted p-values <0.05 were considered statistically significant. To assess differences in cell type composition across experimental conditions, we applied scProportionTest package (33). This approach uses permutation-based framework to test whether observed differences in cluster frequencies exceed those expected by random sampling. Briefly, for each pairwise comparison, we (1) calculated observed log2 fold differences in cluster proportions (2), permuted cell labels across conditions (1,000 iterations) to generate a null distribution, derived two-sided permutation p-values with Benjamini-Hochberg correction to control the FDR, and (3) obtained non-parametric 95% bootstrap confidence intervals for log2 fold differences via bootstrap resampling of cell identities within each condition. To further evaluate the condition-dependent effects on cell subtype populations, we considered both raw cell counts per subtype (as presented in **Supplementary Table 3**; **Supplementary Figures 2**–**6**) and normalized subcluster proportions within parent clusters, visualized through UMAP and bar plots in the main figures. Statistical comparisons of all major cell clusters and subclusters across the four groups of mice are presented in **Supplementary Figure 7**. All statistical analyses related to the scRNA-seq analysis were performed in R (v4.4.2; RRID: SCR_001905). Statistical comparisons for RT-qPCR, ELISA, and histopathological analyses were conducted using two-sided Welch’s *t*-tests (for two-group comparisons) or one-way analysis of variance (ANOVA) followed by Bonferroni’s *post hoc* tests (for four-group comparisons), as specified in the figure legends. p-values <0.05 were considered statistically significant. All statistical analyses were performed in GraphPad Prism (v10.6.0; RRID: SCR_002798).

### 2.15 Graphical illustrations

Graphical illustrations were created using images from NIH BioArt source (https://bioart.niaid.nih.gov) and BioIcons (https://bioicons.com/).

## 3 Results

### 3.1 High-resolution scRNA-seq uncovers cellular heterogeneity in the murine lung microenvironment

To investigate the effect of HDM exposure and IL-1β signaling on the lung immune microenvironment, we treated wild-type (WT) and *Il1b^–/–^* mice on the C57BL/6 background with i.n. administrations of HDM extract or the control vehicle (VEH; 0.9% sodium chloride solution). Following initial sensitization and a one-week rest period, mice were challenged with HDM or VEH three times per week for an additional four weeks. Twenty-four hours after the final i.n. challenge, lungs were harvested, dissociated into single-cell suspensions, and live cells were isolated by fluorescence-activated cell sorting (FACS) before being loaded in duplicate onto the 10x Genomics Chromium platform (**Supplementary Figure 1**). Quality control (QC) metrics and filtering thresholds are provided in **Supplementary Table 3** and further detailed in the Materials and Methods section. After filtering, 51,139 high-quality single cells were retained for downstream analysis.

**Figure 1:**
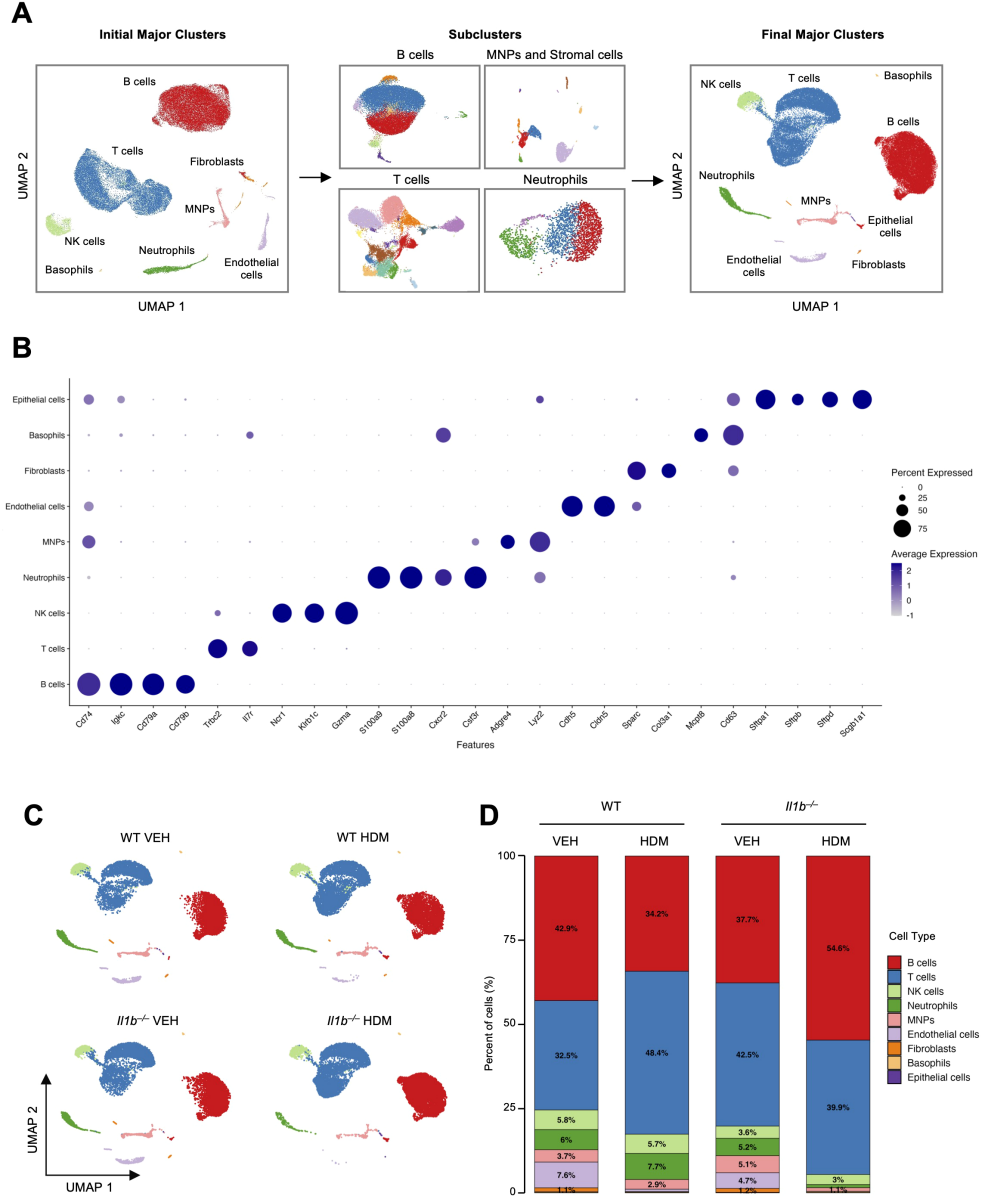
scRNA-seq analysis identifies nine major cell types in the murine lung microenvironment. **(A)** Left: UMAP showing eight initial clusters from pooled lung samples: B cells, T cells, NK cells, neutrophils, mononuclear phagocytes (MNPs), fibroblasts, endothelial cells, and basophils. Middle: Subclustering of B cells, T cells, neutrophils, and MNPs and stromal cells (endothelial cells and fibroblasts) to improve resolution. Right: Final UMAP with nine major cell types after reintegration: B cells, T cells, natural killer (NK) cells, neutrophils, MNPs, endothelial cells, fibroblasts, basophils, and epithelial cells. **(B)** Feature plots for representative marker genes used to annotate each major cell cluster. **(C)** UMAPs of the nine major clusters by genotype and treatment: wild-type (WT) or *Il1b^–/–^* mice treated with vehicle (VEH) or house dust mite (HDM). **(D)** Proportional representation of major cell types across conditions. Cell types comprising <2% of total cells are not labeled.

We integrated the scRNA-seq data from four experimental groups: WT mice treated with VEH (WT VEH) or HDM (WT HDM), and *Il1b^–/–^* mice treated with VEH (*Il1b^–/–^* VEH) or HDM (*Il1b^–/–^* HDM) to identify cluster-specific gene signatures and annotate cell types across conditions. Major cell clusters were identified using Uniform Manifold Approximation and Projection (UMAP) as implemented in the Seurat package (17), and annotated based on canonical marker gene expression, differentially expressed genes (DEGs), and reference-based mapping with SingleR (22). This approach yielded eight initial clusters: B cells, T cells, natural killer (NK) cells, neutrophils, mononuclear phagocytes (MNPs), endothelial cells, fibroblasts, basophils, and epithelial cells (**Figure 1A**).

B cells and neutrophils were individually subclustered to improve resolution and subtype classification, given their sufficient cell numbers. MNPs and stromal cell populations, as well as T and NK cells were grouped based on transcriptional similarity and then subclustered (**Figure 1A**, **Supplementary Figure 2**). Minor cell clusters exhibiting high ribosomal RNA content or transcriptomic profiles suggestive of technical background were further excluded to ensure accurate downstream analysis and cell type annotation. In total, 39 transcriptionally distinct subtypes were identified, each exhibiting gene expression profiles consistent with known functional identities. These subclusters were then consolidated into nine final major cell clusters: B cells (*Cd74, Igkc, Cd79a, Cd79b*), T cells (*Trbc2, Il7r*), NK cells (*Ncr1, Klrb1c, Gzma)*, neutrophils (*S100a9, S100a8, Cxcr2, Csf3r)*, MNPs (*Adgre4, Lyz2)*, endothelial cells (*Cdh5, Cldn5, Vwf)*, fibroblasts (*Sparc, Col3a1)*, basophils (*Mcpt8, Cd63)*, and epithelial cells (*Sftpa1, Sftpb, Sftpd, Scgb1a1)* (**Figures 1A, B**, **Supplementary Figure 2**).

Significant differences in cell-type abundances were observed across conditions, suggesting that both HDM exposure and IL-1β signaling substantially changed the lung cellular composition (**Figures 1C, D**, **Supplementary Figures 7A, B**). Compositional analysis using the scProportionTest package (33) revealed that HDM treatment significantly reduced the relative proportions of fibroblasts and endothelial cells in both WT and *Il1b^–/–^* mice (**Supplementary Figures 7A, C**). In *Il1b^–/–^* HDM, we also observed significantly decreased proportions of neutrophils and MNPs relative to WT HDM and *Il1b^–/–^* VEH (**Supplementary Figures 7C, D**). Conversely, B cell abundance was increased in *Il1b^–/–^* HDM compared to WT HDM (**Supplementary Figure 7D**). Although basophils and epithelial cells were captured and included in the dataset, their low abundance across all groups limited the statistical power of compositional analyses; therefore, they were not further discussed in the Results section (**Supplementary Figures 2, 7C**).

### 3.2 HDM exposure and IL-1β signaling differentially affect B cell activation and differentiation in the lungs

**Figure 2:**
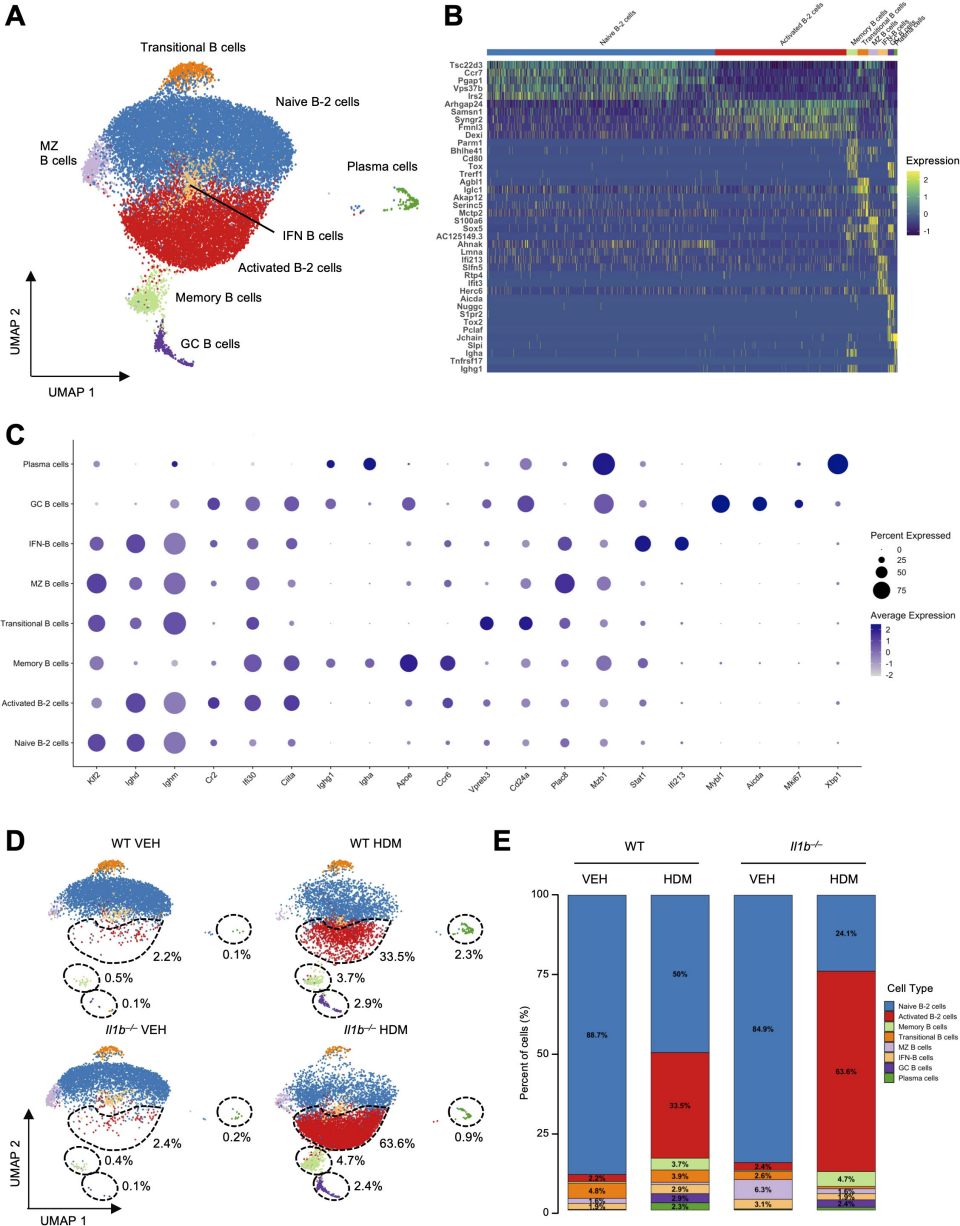
The impact of HDM exposure and IL-1β signaling on B cell subclusters. **(A)** UMAP of the eight B cell subclusters: naive B-2 cells, activated B-2 cells, memory B cells, transitional B cells, marginal zone B cells (MZ B cells), interferon-activated B cells (IFN B cells), germinal center B cells (GC B cells), and plasma cells. **(B)** Heatmap of the top five DEGs per subtype, illustrating distinct transcriptomic profiles. Gene expression is scaled by Log2 fold change. **(C)** Dot plot showing expression of canonical and subtype-specific marker genes across B cell clusters. **(D)** UMAPs of the eight B cell subclusters across the four experimental conditions. Black dotted circles highlight the modulation of activated B-2 cells, GC B cells, memory B cells, and plasma cells across the different conditions. The relative percentages of these populations in each treatment group are indicated. **(E)** Proportional representation of B cell subtypes across conditions. Subtypes comprising of <2% of total B cells are not labelled.

The largest population of cells (22,085 cells; accounting for 43.19% of all cells in the pooled samples) was identified as B cells, characterized by expression of B cell antigen receptor genes (*Cd79a, Cd79b*), the MHC II encoding gene (*Cd74*), and the immunoglobulin gene (*Igkc*) (**Figure 1B**, **Supplementary Figure 2**). The B cell cluster was further subclustered into 8 distinct subtypes to improve resolution. Annotation was based on UMAP clustering, DEGs, canonical marker gene expression, and transcriptomic profiles reported in previous studies, as follows: naive B-2 cells (*Klf2, Ccr7, Ighd, Ighm*), activated B-2 cells (*Cr2, Ciita, Samsn1, Ifi30*), memory B cells (*Apoe, Ighg1, Igha, Ccr6, Cd80*), transitional B cells (*Iglc1, Vpreb3, Cd24a*), marginal zone B cells (MZ B cells; *Plac8, Mzb1, Ighd, Ighm*), B cells with high interferon-stimulated gene expression (IFN B cells; *Stat1, Ifi27l2a, Ifit3, Isg15*), germinal center B cells (GC B cells; *Aicda, Mki67, Myb1, Pclaf*), and plasma cells (*Jchain*, *Xbp1*, *Igha, Ighg1*, *Slpi*) (**Figures 2A–C**, **Supplementary Figure 3**) (34–39).

Naive B-2 cells (also called conventional follicular B cells) showed a significant reduction in HDM-treated groups, decreasing from 4,266 to 2,080 cells (88.7% to 50%) in WT, and from 3,866 to 2,068 cells (84.9% to 24.1%) in *Il1b^–/–^* mice (**Figure 2D, E****,****Supplementary Figures 3**, **7E, G**). Similarly, the absolute numbers and relative proportions of transitional B cells and MZ B cells were decreased in both WT HDM from 233 to 162 cells (4.8% to 3.9%) for transitional B cells and from 77 to 29 cells (1.6% to 0.7%) for MZ B cells, and *Il1b^–/–^* HDM from 117 to 66 cells (2.6% to 0.8%) for transitional B cells and from 285 to 133 cells (6.3% to 1.6%) for MZ B cells (**Figures 2D, E**, **Supplementary Figures 3**, **7E, G**). These data suggest that naive B-2 cells, transitional B cells, and MZ B cells may contribute to maintaining immune quiescence and initiating specific humoral responses against HDM. This could occur either through activation and differentiation into other B cell subsets or via apoptosis following strong antigen engagement (40, 41).

Consistent with this hypothesis, several activated and differentiated B cell subsets were increased in both WT HDM and *Il1b^–/–^* HDM groups. Notably, activated B-2 cells expanded from 108 to 1396 cells (2.2% to 33.5%) in WT mice and from 111 to 5,451 cells (2.4% to 63.6%) in *Il1b^–/–^* mice, memory B cells expanded from 23 to 156 cells (0.5% to 3.7%) in WT mice and from 17 to 402 cells (0.4% to 4.7%) in *Il1b^–/–^* mice, GC B cells expanded from 5 to 121 cells (0.1% to 2.9%) in WT and from 5 to 203 cells (0.1% to 2.4%) in *Il1b^–/–^* mice, and plasma cells expanded from 4 to 95 cells (0.1% to 2.3%) in WT and from 11 to 75 cells (0.2% to 0.9%) in *Il1b^–/–^* mice, further supporting the activation of humoral immunity in response to chronic HDM exposure (**Figures 2D, E**, **Supplementary Figures 3**, **7E–H**). Among these subsets, the numbers of activated B-2 cells, GC B cells, and memory B cells were significantly higher in *Il1b^–/–^* HDM than WT HDM, suggesting a potential compensatory B cell response to chronic HDM exposure in the absence of IL-1β (**Supplementary Figure 7H**). However, the baseline numbers of most B cell subtypes, except for transitional B cells and MZ B cells, were not significantly different between WT and *Il1b^–/–^* VEH groups, suggesting that IL-1β does not significantly affect B cell populations under homeostatic conditions (**Supplementary Figure 7F**). IFN B cells, characterized by high IFN-stimulated gene expression, showed a modest increase in absolute numbers following HDM exposure in both WT (from 92 to 122 cells) and *Il1b^–/–^* mice (from 139 to 167 cells). Their relative proportions slightly increased in WT HDM mice (from 1.9% to 2.9%) but declined in *Il1b*^-^/^-^ HDM mice (from 3.1% to 1.9%), suggesting that although IFN B cells accumulate in response to HDM, their expansion may be outpaced by other B cell subsets, particularly when IL-1β signaling is absent.

To further investigate the role of IL-1β in B cell activation and differentiation in response to HDM, we performed comprehensive analyses on activated B-2 cells, including DEG analysis, transcription factor enrichment analysis (TFEA), pathway enrichment analysis, and gene ontology (GO) analysis. In WT mice, HDM exposure induced robust activation of a stress-response and NF-κB-associated transcription factors, including *Hsf2, Nfat5, Gata1, Hsf1, Nfyc, Xbp1*, and *Nfyb*, along with enrichment of NF-κB pathway (**Supplementary Figures 8A, B**). In contrast, *Il1b^–/–^* HDM mice showed upregulation of *Gata1* but lacked strong activation of the HSF-family and NFY complex, and instead displayed enrichment of Tumor Necrosis Factor (TNF), TNF-Related Apoptosis-Inducing Ligand (TRAIL), Vascular Endothelial Growth Factor (VEGF), and Phosphoinositide 3-Kinase (PI3K) pathways, suggesting compensatory signaling mechanisms to maintain B cell proliferation and activation in the absence of IL-1β during chronic HDM exposure (**Supplementary Figures 8C, D**).

Direct comparison of WT and *Il1b^–/–^* HDM groups revealed that activated B-2 cells induced in response to chronic HDM exposure exhibited higher TF activity of *Hsf1/2*, *Nfyc, Nfyb, Nfat5, Klf4, Ddit3, Nfya, Foxo3, and Foxa1*, alongside enrichment of Nuclear Factor kappa-light-chain-enhancer of activated B cells (NF-κB), androgen, Transforming Growth Factor Beta (TGF-β), Mitogen-Activated Protein Kinase (MAPK), and Tumor Protein p53 (p53) signaling pathways in presence of IL-1β (**Supplementary Figure 8E**). In contrast, activated B-2 cells in *Il1b^–/–^* HDM mice showed stronger activation of the TNF, TRAIL, VEGF, and WNT pathways, with GO enrichment indicating high cell proliferation and B cell activation (**Supplementary Figures 8F, G**). These results suggest that HDM exposure induces a stress-response and NF-κB-associated transcriptional program influenced by IL-1β signaling, whereas in its absence, B cells activate alternative pathways to sustain humoral responses.

No significant DEGs were observed in activated B-2 cells between WT and *Il1b^–/–^* VEH groups, suggesting that IL-1β primarily influences B cell responses during HDM exposure rather than under homeostatic conditions (**Supplementary Figure 8H**). Similar enrichment patterns between WT and *Il1b^–/–^* HDM groups were observed across other B cells subtypes (**Supplementary Figure 9**), suggesting that HDM-induced transcriptomic programs and compensatory gene regulatory mechanisms in the absence of IL-1β are not limited to activated B-2 cells.

### 3.3 HDM exposure, and to a lesser extent IL-1β signaling, modulates various T cell populations in the lungs

**Figure 3:**
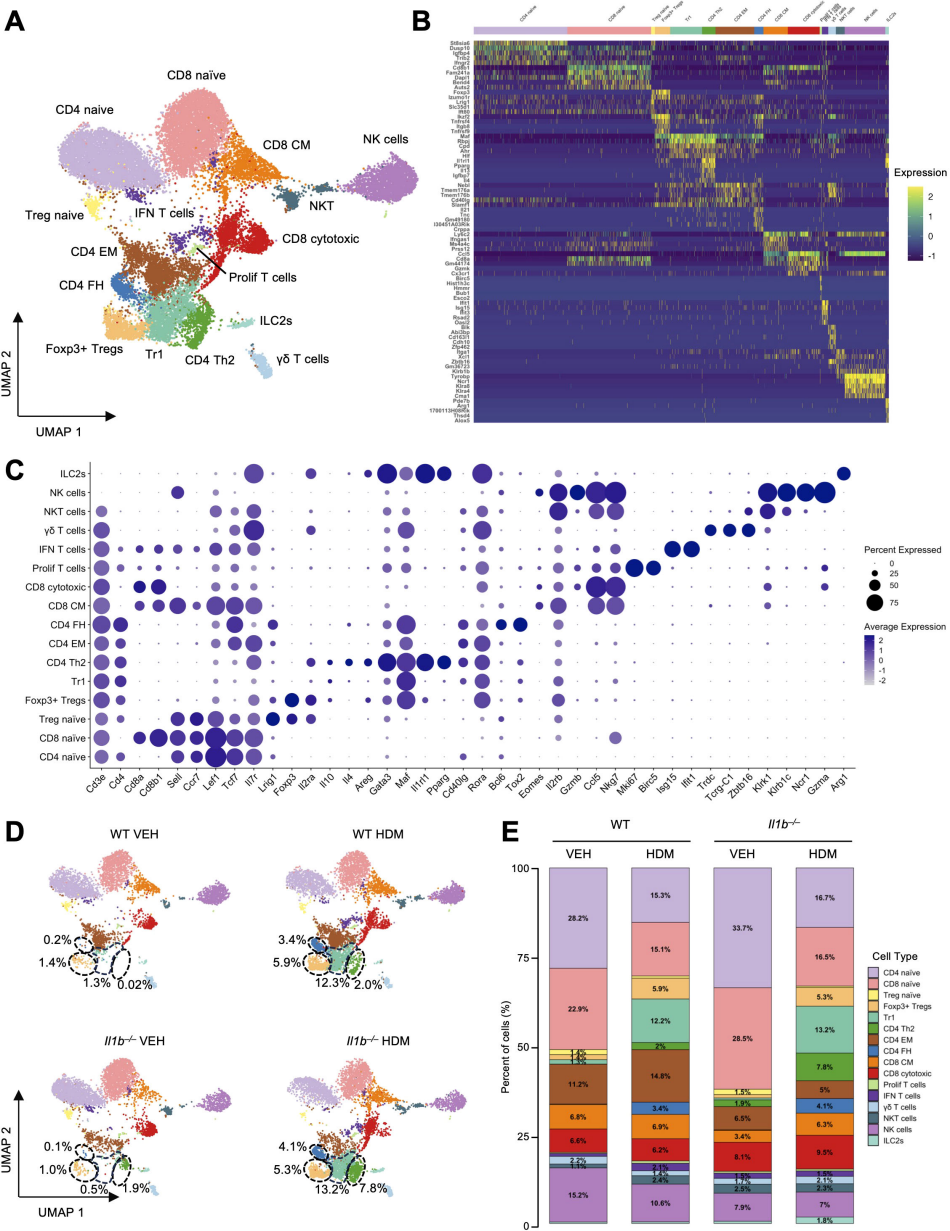
The impact of HDM exposure and IL-1β signaling on T and NK cell subclusters. **(A)** UMAP of the fifteen T cell subclusters: naive CD4 T cells (CD4 naive), naive CD8 T cells (CD8 naive), natural killer cells (NK cells), effector memory CD4 T cells (CD4 EM), cytotoxic CD8 T cells (CD8 cytotoxic), type 1 regulatory T cells (Tr1), central memory CD8 T cells (CD8 CM), CD4 Foxp3^+^ regulatory T cells (Foxp3^+^ Tregs), CD4 T helper 2 cells (CD4 Th2), follicular helper CD4 T cells (CD4 FH), natural killer T cells (NKT cells), γδ T cells, interferon-activated CD8 T cells (IFN T cells), naive regulatory T cells (Treg naive), type 2 innate lymphoid cells (ILC2s), and proliferating T cells (Prolif T cells). **(B)** Heatmap of the top five DEGs per subtype, illustrating distinct transcriptomic profiles. Gene expression is scaled by Log2 fold change. **(C)** Dot plot showing expression of canonical and subtype-specific marker genes across T cell subtypes. **(D)** UMAP of the fifteen T cell subclusters across the four experimental conditions. Black dotted circles highlight the modulation of Foxp3^+^ Tregs, Tr1, and CD4 Th2 cells across the different conditions. The relative percentages of these populations in each treatment group are indicated. **(E)** Proportional representation of T cell subtypes across conditions. Cell types comprising of <1% are not labelled.

T cells constituted the second largest population overall (20,922 cells, accounting for 40.91% of all cells in the pooled samples) and were the most abundant population in both WT HDM (5,887 cells, 48.4%) and *Il1b^–/–^* VEH (5,139 cells, 42.5%) groups (**Figure 1C**, **Supplementary Figure 2**). To improve the resolution of cell subtypes, we merged and subclustered the initial T and NK cell clusters (**Figure 1A**), identifying 16 distinct cell subtypes (**Figure 3A**). Based on DEGs and canonical marker gene expression, we annotated the following cell subtypes: CD4 naive T cells (CD4 naive; *Cd4, Ccr7, Sell, Lef1, Tcf7, Il7r*), CD8 naive T cells (CD8 naive; *Cd8a, Cd8b1, Ccr7, Sell, Lef1, Tcf7, Il7r*), NK cells (*Cd3e^–^, Ncr1, Klrb1c, Gzma, Klrk1)*, CD4 effector memory T cells (CD4 EM; *Cd4, Il21, Cd44, Tcf, Maf, Rora, Cd40lg*), CD8 cytotoxic T cells (CD8 cytotoxic; *Cd8a, Cd8b1, Gzmb, Gzmk, Ccl5, Nkg7*), Type 1 regulatory T cells (Tr1; *Cd4, Maf, Rbpj, Ahr, Foxp3^–^, Gata3^low^, Il4^–^*), CD8 central memory T cells (CD8 CM; *Cd8a/b1, Sell, Lef1, Tcf7, Il7r, Eomes, Il2rb, Bcl2, Ccl5, Nkg7, Ly6c2*), Foxp3^+^ CD4 regulatory T cells (Foxp3^+^ Tregs; *Cd4, Foxp3, Ikzf2, Il2ra, Tnfrsf4, Itgb8*), CD4^+^ T helper 2 (CD4 Th2; *Cd4, Maf, Il1rl1, Gata3, Il4, Il13, Pparg)*, CD4 follicular helper T cells (CD4 FH; *Cd4, Tcf7, Lrig1, Maf, Tox2, Il21, Cd40lg*), natural killer T cells (NKT cells; *Cd3e, Itga1, Xcl1, Zbtb16, Klrb1b, Tyrobp, Klrk, Trdc^–^*), γδ T cells (*Cd3e, Cd4^–^, Cd8a^–^/b1^–^, Trdc, Tcrgc1, Zbtb16, Il23r, Il1r1, Il7r, Maf, Rora*), mixed CD4 and CD8 T cells with high interferon-stimulated gene expression (IFN T cells; *Cd3e, Ifit1, Isg15, Rsad2*), naive regulatory T cells (Treg naive; *Foxp3, Sell, Ccr7, Il2ra, Lrig1)*, group 2 innate lymphoid cells (ILC2s; *Cd3e^–^, Rora, Il1rl1, Arg1, Il7r, Gata3*), and T cells with a proliferation gene expression signature (Prolif T cells; *Mki67, Birc5, Cenpf, Top2a, Cdk1*) (**Figures 3A–C**, **Supplementary Figure 4**) (42–47).

In both WT and *Il1b^–/–^* mice, CD4 and CD8 naive were the dominant subsets. Chronic HDM exposure led to a reduction in CD4, CD8, and Treg naive cells and an expansion of differentiated T cell subtypes in both WT and *Il1b^–/–^* mice (**Figures 3D, E**). Notably, Foxp3^+^ Tregs (increasing from 61 to 386 cells, 1.4% to 5.9%, in WT, and from 54 to 355 cells, 1.0% to 5.3%, in *Il1b^–/–^*), Tr1 (increasing from 54 to 806 cells, 1.3% to 12.3%, in WT, and from 30 to 886 cells, 0.5% to 13.2%, in *Il1b^–/–^*), CD8 CM (increasing from 294 to 453 cells, 6.8% to 6.9%, in WT, and from 190 to 422 cells, 3.4% to 6.3%, in *Il1b^–/–^*), and CD4 FH (increasing from 7 to 227 cells, 0.2% to 3.4%, in WT, and from 7 to 275 cells, 0.1% to 4.1%, in *Il1b^–/–^*) were markedly increased in both WT and *Il1b^–/–^* HDM groups, suggesting that the associated T cell immune response may occurs largely independently of IL-1β signaling (**Figures 3D, E**, **Supplementary Figures 4**, **7E, G, H**).

In line with HDM-driven Th2 responses (1), CD4 Th2 cells were significantly expanded in WT HDM mice compared to WT VEH controls, increasing from 1 to 132 cells (0.02% to 2.00%), indicating a Th2 polarization of the lung microenvironment in response to chronic HDM exposure. *Il1b^–/–^* HDM mice also showed a marked increase in CD4 Th2 cells compared to *Il1b^–/–^* VEH controls, rising from 106 to 523 cells (1.9% to 7.8%) (**Figures 3D, E**, **Supplementary Figures 7E, G**). Interestingly, *Il1b^–/–^* mice exhibited higher baseline and HDM-induced CD4 Th2 populations than WT, suggesting a potential regulatory role of IL-1β in limiting Th2 cell expansion. Additionally, ILC2s, which are key Th2-promoting innate lymphoid cells (48), were significantly enriched in *Il1b^–/–^* HDM compared to both *Il1b^–/–^* VEH and WT HDM, increasing from 17 to 29 cells (0.02% to 0.44%) in WT, and from 35 to 120 cells (0.6% to 1.8%) in *Il1b^–/–^* mice, further reinforcing the Th2-biased immune response induced by HDM exposure in the absence of IL-1β.

In contrast, CD4 EM were markedly expanded in WT HDM (from 481 to 972 cells, 11.2% to 14.8%), but not in *Il1b^–/–^* HDM mice, where their numbers slightly decreased (from 365 to 337 cells, 6.5% to 5.0%), suggesting that this subcluster is modulated by IL-1β in response to HDM. Notably, the top expressed genes in CD4 EM included *Tmem176a* and *Tmem176b*, indicating a Th17-like phenotype (49) and supporting IL-1β’s established role in promoting Th17 differentiation (50, 51). DEG analysis of CD4 EM between WT HDM and *Il1b^–/–^* HDM mice revealed enrichment of Th17 cell differentiation pathways in KEGG, increased *Rora* and *Rorc* transcription factor (TF) activity, and enhanced TGF-β pathway activity in PROGENy analysis, collectively confirming a Th17-promoting effect of HDM in the presence of IL-1β (**Supplementary Figures 10A–C**) (52–54). Supporting this, TF activity analysis of CD4 EM showed higher *Rora* and *Rorc* activity in WT VEH compared to *Il1b^–/–^* VEH, suggesting a baseline Th17 bias mediated by IL-1β (**Supplementary Figure 10D**). Upon HDM treatment, only WT HDM showed upregulation of JAK-STAT signaling, a known inducer of STAT3, which promotes Th17 differentiation (55), whereas *Il1b^–/–^* HDM exhibited increased MAPK activity alone (**Supplementary Figures 10E, F**). These results further highlight the role of IL-1β in promoting Th17 cell proliferation and activation during chronic HDM exposure.

Smaller populations, including CD4 and CD8 T cells expressing interferon-stimulated genes (IFN T cells), NKT cells, and Prolif T cells exhibited higher baseline levels in *Il1b^–/–^* mice (81, 141, and 28 cells; 1.5%, 2.5%, and 0.5%) compared to WT (35, 46, and 13 cells; 0.8%, 1.1%, and 0.3%). However, these subsets were significantly increased in WT HDM mice (138, 155, and 44 cells; 2.1%, 2.4%, and 0.7%), but not in *Il1b^–/–^* HDM mice (101, 157, and 37 cells; 1.5%, 2.3%, and 0.6%), suggesting that IL-1β may negatively regulate the expansion or maintenance of these subsets under steady-state conditions. Other cell populations, including γδ T cells, NK cells, CD8 cytotoxic T cells were not significantly affected by either HDM exposure or IL-1β signaling.

### 3.4 HDM exposure triggers IL-1β-driven neutrophil activation

**Figure 4:**
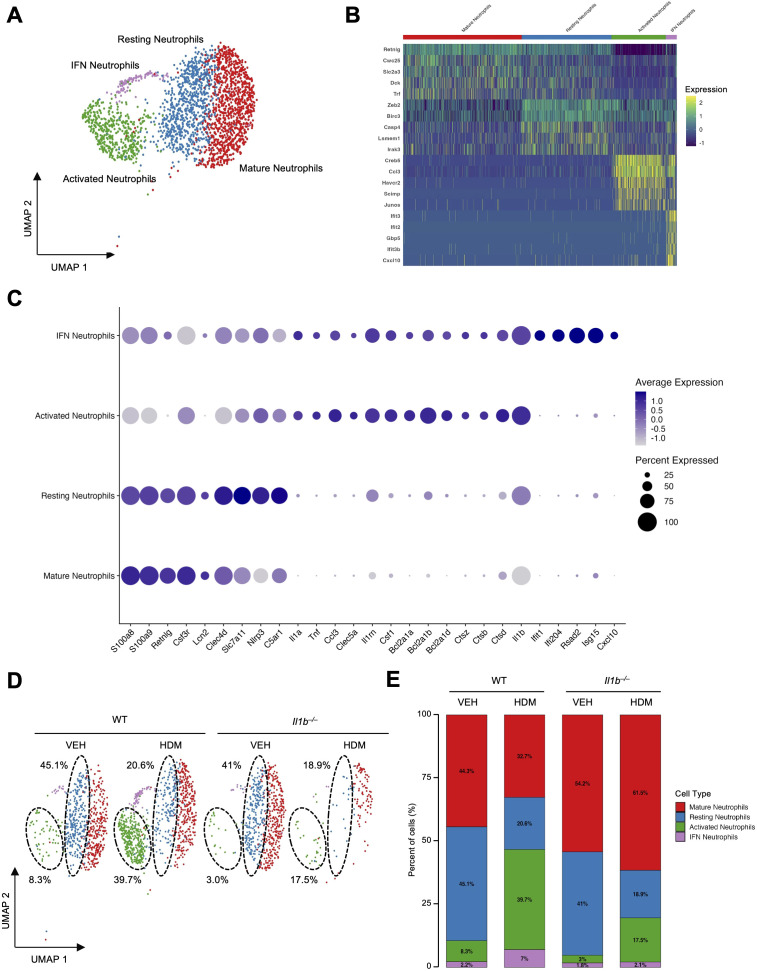
The impact of HDM exposure and IL-1β signaling on neutrophil subclusters. **(A)** UMAP of the four neutrophil subsets: mature neutrophils, resting neutrophils, activated neutrophils, and interferon-activated neutrophils (IFN neutrophils). **(B)** Heatmap of the top five DEGs per subtype, illustrating distinct transcriptomic profiles. Gene expression is scaled by Log2 fold change. **(C)** Dot plot showing expression of canonical and subset-specific marker genes across neutrophil subtypes. **(D)** UMAP of the four neutrophil subclusters across the four experimental conditions. Black dotted circles highlight the modulation of resting and activated neutrophils across the different conditions. The relative percentages of these populations in each treatment group are indicated. **(E)** Proportional representation of neutrophil subtypes across conditions.

The number of neutrophils (2,441 cells; accounting for 4.73% of all cells in the pooled samples) increased in WT HDM compared to WT VEH mice (from 672 to 940 cells, 6.0% to 7.7%), but was significantly reduced in *Il1b^–/–^* HDM (143 cells, 0.9%), despite comparable numbers in *Il1b^–/–^* VEH (625 cells, 5.2%) (**Figures 1A–D**, **Supplementary Figures 2**; **7A–D**). This difference suggests that IL-1β is required for neutrophil recruitment during chronic HDM exposure, consistent with previous reports showing reduced neutrophil infiltration into tissues in *Il1b^–/–^* mice during inflammatory responses (56, 57). Given the significant impact of HDM exposure and IL-1β signaling on neutrophil absolute numbers and relative proportions, we performed subclustering and identified four distinct neutrophil populations based on DEGs and canonical marker gene expression: mature neutrophils (*S100a8, S100a9, Retnlg, Csf3r, Lcn2*), resting neutrophils (*Clec4d, Slc7a11, Nlrp3, C5ar1*), activated neutrophils (*Il1a, Tnf, Ccl3, Clec5a, Il1rn*), and neutrophils with high interferon-stimulated gene expression (IFN neutrophils; *Ifit1, Ifi204, Rsad2, Isg15, Cxcl10*) (**Figures 4A–C**, **Supplementary Figure 5**) (58).

The absolute numbers and relative proportions of resting and mature neutrophils were relatively unaffected by IL-1β under baseline conditions, as shown in WT VEH and *Il1b^–/–^* VEH groups (**Figures 4D, E**, **Supplementary Figures 5**, **7F**). However, the absolute numbers and relative proportions of resting neutrophils were decreased in WT HDM (from 303 to 194 cells, 45.1% to 20.6%) and *Il1b^–/–^* HDM groups (from 256 to 27 cells, 41.0% to 18.9%) (**Figures 4D, E**, **Supplementary Figures 5**, **7E, G**). In contrast, activated neutrophils, and to a lesser degree, IFN neutrophils, were significantly increased in WT HDM compared to WT VEH, and this effect was largely abrogated in *Il1b^–/–^* mice (**Figures 4D, E**, **Supplementary Figures 5**, **7E–H**). This suggests an IL-1β-mediated recruitment and activation of neutrophils in response to HDM. DEG analysis of activated neutrophils revealed significant upregulation of genes involved in chemotaxis and inflammation, including *Ccl3, Tnf, Csf1, Il1a, Il1b*), as well as anti-apoptotic genes (*Bcl2a1a, Bcl2a1b, Bcl2a1d*) and lysosomal cysteine proteases-encoding genes (*Ctsz, Ctsb, Ctsd, Ctss*). These findings suggest an IL-1β-dependent, neutrophil-mediated immune response against HDM (**Figures 4B, C**). The marked reduction of all neutrophil subsets in *Il1b^–/–^* HDM mice further support the essential role of IL-1β in neutrophil recruitment, activation, and survival under inflammatory conditions, consistent with previous reports on IL-1β-driven neutrophil regulation (57).

*Il1b* was strongly expressed (average log fold ~4.3-4.5) in both activated neutrophil and IFN neutrophil subsets (**Figure 4C**). Although *Il1b* and *Nlrp3* transcripts levels within these subsets were not significantly increased by HDM treatment under our experimental conditions, the HDM-induced expansion of activated and IFN neutrophils observed in WT mice likely contributes to the overall increase in *Il1b* expression observed in the lungs.

### 3.5 HDM exposure induces the activation and differentiation of distinct myeloid cell types via both IL-1β-dependent and -independent pathways

**Figure 5:**
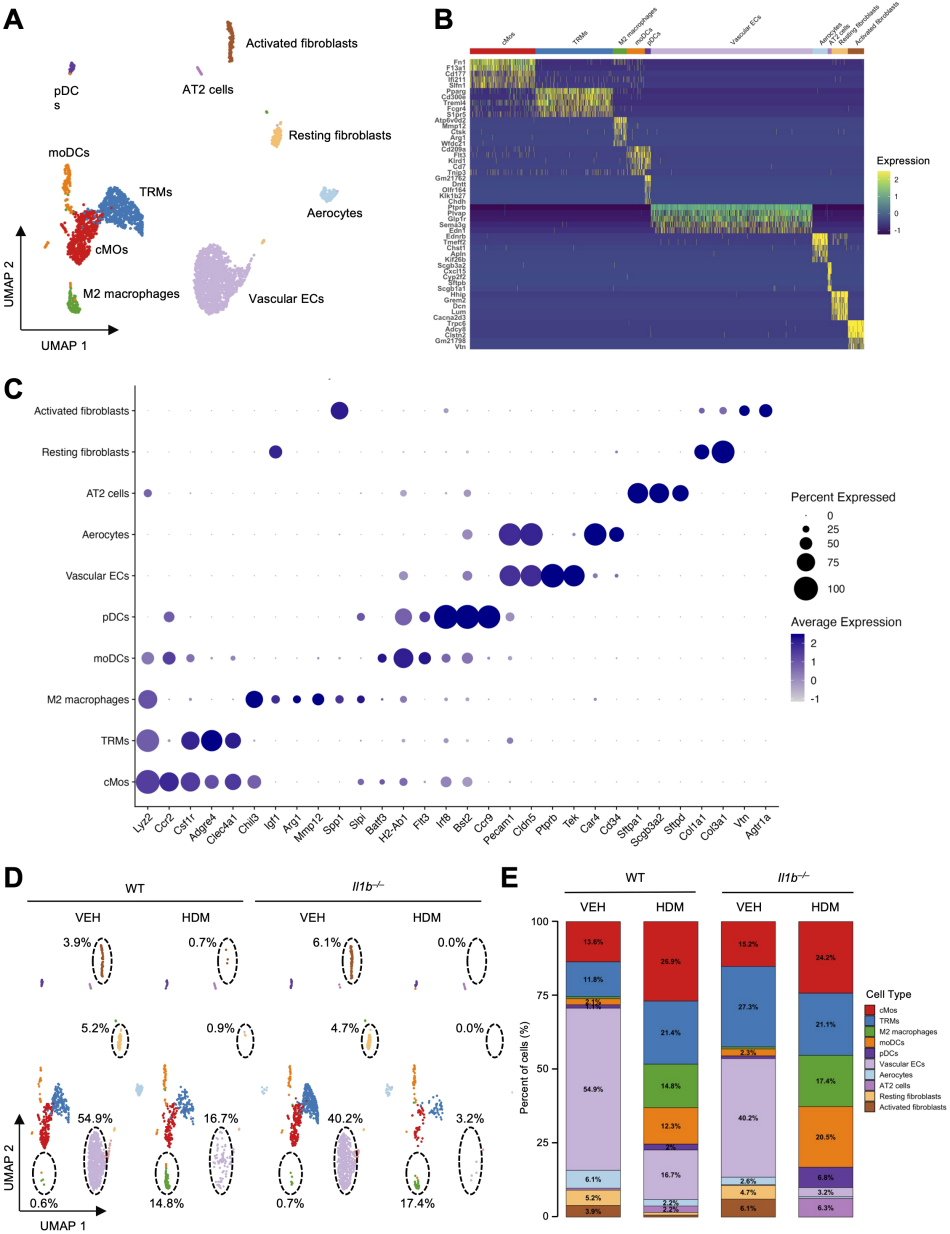
The impact of HDM exposure and IL-1β signaling on MNPs and stromal cells. **(A)** UMAP of the eleven MNP and stromal cell subsets: vascular endothelial cells (Vascular ECs), tissue-resident macrophages (TRMs), classical monocytes (cMos), monocyte-derived dendritic cells (moDCs), resting fibroblasts, activated fibroblasts, aerocytes, alternative-activated macrophages (M2 macrophages), plasmacytoid dendritic cells (pDCs), and alveolar type II cells (AT2 cells). **(B)** Heatmap of the top five DEGs per subtype, illustrating distinct transcriptomic profiles. Gene expression is scaled by Log2 fold change. **(C)** Dot plot showing expression of canonical and subset-specific marker genes across MNP and stromal cell subtypes. **(D)** UMAP of the eleven MNP and stromal cell subclusters across the four experimental conditions. Black dotted circles highlight the modulation of resting and activated fibroblasts, M2 macrophages, and vascular ECs across the different conditions. The relative percentages of these populations in each treatment group are indicated. **(E)** Proportional representation of the MNP and stromal cell subtypes across conditions.

To enhance the resolution of the remaining captured cell types, we merged and subclustered the initial clusters of MNPs and stromal cells, yielding the following 5 MNP and 6 stromal cell subclusters: vascular endothelial cells (Vascular ECs; *Pecam, Cldn5, Ptprb, Tek*), classical monocytes (cMos; *Ccr2, Csf1r, Lyz2, Fn1, F13a1, Mafb, Csf1r*), tissue resident macrophages (TRMs; *Lyz2, Adgre4, Pparg, Clec4a1, Cd300e*), monocyte-derived dendritic cells (moDCs; *Lyz2, Ccr2, Fn1, F13a1, Mafb, Csf1r*), resting fibroblasts (*Dcn, Lum, Gbp, Hhip, Igfbp5*), and activated fibroblasts (*Postn, Trpc6, Adcy8*), aerocytes (Ednrb, Emp2, Car4), alternatively activated macrophages (M2 macrophages; *Chil3, Arg1, Igf1*, *Mrc1, Fabp4*), plasmacytoid dendritic cells (pDCs; *siglech, Tcf4, Bst2, Irf8, Grm8*), and alveolar type II cells (AT2 cells; *Stfpa1, Sftpb, Sftpd, Cbr2, Scgb1a1*) (**Figures 5A–C**, **Supplementary Figure 6**).

cMos, which serve as precursors for macrophages or dendritic cells upon entering the lung, were reduced in absolute numbers following HDM treatment in both WT and *Il1b^–/–^* mice, with a more pronounced decrease in the *Il1b^–/–^* group (from 191 to 122 cells in WT and from 200 to 46 cells in *Il1b^–/–^* mice) (**Supplementary Figures 6**, **7E, G**). However, their relative proportions increased in both WT (from 13.6% to 26.9%) and *Il1b^–/–^* mice (from 15.2% to 24.2%) following HDM exposure (**Figures 5D, E**), likely reflecting a more substantial reduction in other immune cell populations. These changes suggest that by approximately 24 hours after the final HDM exposure, many infiltrating monocytes may have already differentiated into downstream populations such as M2 macrophages or moDCs.

In line with this hypothesis, the absolute numbers of M2 macrophages were increased following HDM treatment in both WT (from 9 to 67 cells) and *Il1b^–/–^* mice (from 9 to 33 cells), with their relative proportions rising more substantially from 0.6% to 14.8% in WT and from 0.7% to 17.4% in *Il1b^–/–^* mice (**Figures 5D, E**, **Supplementary Figures 6**, **7E, G, H**). Differential expression analysis of M2 macrophages revealed robust upregulation of genes associated with tissue remodeling, anti-inflammatory responses, and IL-1β regulation, including *Arg1, Igf1, Mmp12, Spp1, Il1rn*, and *Slpi*, highlighting the distinct role of M2 macrophages in the lung microenvironment during chronic HDM exposure (59–63) (**Figures 5B, C**). Similarly, while the absolute number of moDCs increased modestly following HDM treatment in WT (from 29 to 56 cells) and in *Il1b^–/–^* mice (from 31 to 39 cells), their relative percentages rose more substantially from 2.1% to 12.3% in WT and from 2.3% to 20.5% in *Il1b^–/–^* mice (**Figures 5D, E**, **Supplementary Figures 6**, **7E, G, H**), likely reflecting changes in the overall cellular composition and reduced total cell recovery, particularly in the *Il1b^–/–^* HDM group. These changes, along with the observed decrease in cMos and increase in M2 macrophages, suggest that both moDCs and M2 macrophages may have differentiated from cMos in response to HDM exposure, potentially through both IL-1β-dependent and -independent pathways.

Interestingly, *Il1b^–/–^* VEH exhibited elevated TRM numbers compared to WT VEH. However, TRM populations declined sharply following HDM exposure in both WT and *Il1b^–/–^* mice (from 166 to 97 cells in WT and from 360 to 40 cells in *Il1b^–/–^* mice). In addition, while the relative proportion of TRMs increased from 11.8% to 21.4% in WT mice, it slightly decreased from 27.3% to 21.1% in *Il1b^–/–^* mice (**Figures 5D, E**, **Supplementary Figures 6**, **7E–H**). Given the established role of TRMs in maintaining lung homeostasis through apoptotic cell clearance and immune regulation, these findings suggest that IL-1β deficiency may lead to an accumulation of TRMs under steady-state conditions, but either impairs their function or increases their susceptibility to apoptosis during chronic inflammation.

A marked reduction in both absolute numbers and relative proportions of vascular ECs was observed was observed in the lungs of both WT (from 771 to 76 cells, 54.9% to 16.7%) and *Il1b^–/–^* mice (from 530 to 6 cells, 40.2% to 3.2%) following HDM exposure (**Figures 5D, E**, **Supplementary Figures 6**, **7E–H**). This reduction may result from inflammation-induced endothelial damage, vascular rarefaction, or endothelial-to-mesenchymal transition (EndMT), which are processes commonly triggered by chronic allergic inflammation (64). Indeed, DEG and TF analyses revealed increased enrichment of heat shock protein (HSP) family genes (*Hspa1a*, *Hspa1b, Hsph1)*, elevated hypoxia pathway activity, and upregulation of angiogenesis-related pathways (WNT, VEGF, EGFR) in WT HDM compared to WT VEH (**Supplementary Figure 11**). These findings suggest potential HDM-induced endothelial damage and subsequent angiogenic responses (65). Additionally, technical limitations such as poor recovery of fragile endothelial cells during tissue dissociation and reduced marker gene expression under inflammatory stress may also contribute to their underrepresentation in our dataset. Similar factors may explain the marked reduction in both the absolute numbers and relative proportions of resting and activated fibroblasts observed in the lungs of WT and *Il1b^–/–^* mice following HDM exposure (**Figures 5D, E**, **Supplementary Figures 6**, **7E–H**). Aerocytes, a recently identified lung-specific endothelial cell subset specialized in gas exchange and leukocyte trafficking (66), were also markedly reduced in HDM-treated groups compared to VEH-treated controls (decreasing from 85 to 10 cells in WT and from 34 to 1 cells in *Il1b^–/–^* mice) (**Figure 5D**, **Supplementary Figures 5**, **7E, G**). Lastly, AT2 cells (the only epithelial subset identified) and pDCs were not captured in sufficient numbers to enable a robust assessment of HDM exposure or IL-1β signaling. Collectively, these scRNA-seq findings indicate that HDM exposure remodels the lung immune landscape by modulating multiple lymphoid and myeloid cell subsets through both IL-1β-dependent and -independent mechanisms. Moreover, HDM exposure may reduce the viability of specific immune or stromal cell populations, either as a result of inflammation-driven cell loss or technical factors affecting cell recovery.

### 3.6 Validation of scRNA-seq findings by histopathology, cytokine profiling, and immunofluorescence analysis

**Figure 6:**
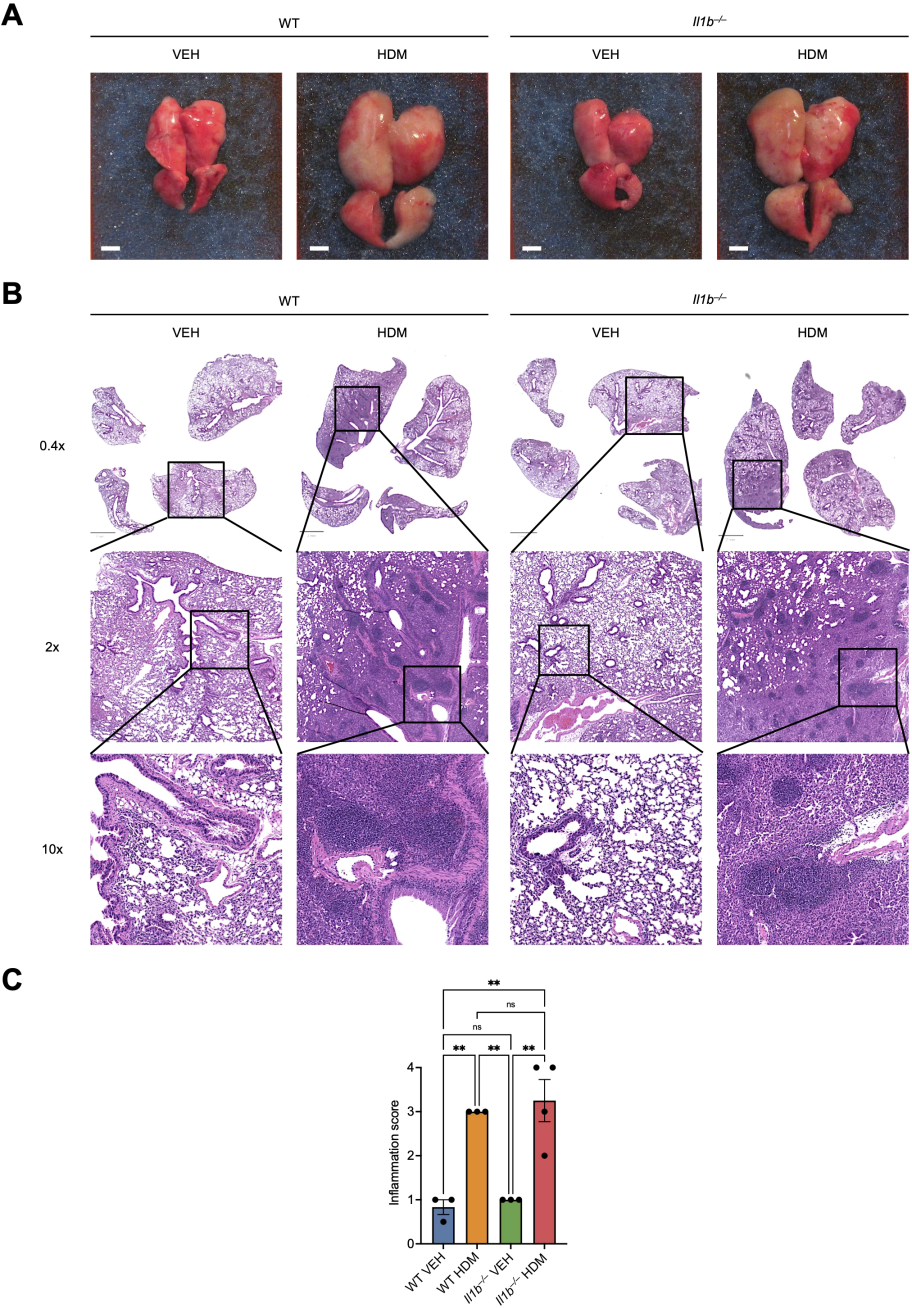
Histopathological analysis of the lungs. **(A)** Representative photos of four lung lobes (dorsal view) of WT and *Il1b^–/–^* mice treated with VEH or HDM as shown in **Figure 1A** (n = 3–4 mice per group; scale bars = 0.25 cm). Cardiac perfusion resulted in a color change to pink/white. **(B)** Representative photos of H&E-stained lung sections. The top panels show four lung lobes from one mouse per group (scale bars = 2 mm). The lower panels display the boxed regions at higher magnification, highlighting diffuse mixed inflammatory infiltrates in peribronchiolar and perivascular regions of WT and *Il1b^–/–^* mice treated with HDM, whereas VEH-treated mice exhibit minimal inflammatory changes and similar patterns of alveolar collapse, likely attributable to tissue harvesting artifacts. **(C)** Quantification of inflammatory cell infiltrates in H&E-stained lung sections shown in **(B)**. Data are presented as mean ± SEM. Statistical significance was determined by one-way ANOVA with *post hoc* Bonferroni’s test. ns, not significant; **p < 0.01.

To validate key findings from the scRNA-seq analysis, we treated an independent cohort of mice as shown in **Figure 1A** and first performed a histopathological evaluation of the lungs across the four experimental groups. Gross examination revealed that both WT and *Il1b^–/–^* mice treated with HDM developed lung inflammation, as indicated by the enlarged lungs compared to those of mice VEH-treated mice (**Figure 6A**). In addition, H&E staining and histopathological analysis revealed that both WT and *Il1b^–/–^* mice treated with HDM developed diffuse mixed inflammatory infiltrates in peribronchiolar and perivascular areas, while both VEH groups display minimal inflammatory changes (**Figure 6B**). Inflammation scoring confirmed the significant increase in inflammatory cell infiltrates in the lungs of both WT HDM and *Il1b^–/–^* HDM, whereas these inflammatory features were essentially absent in the lungs of *Il1b^–/–^* VEH (**Figure 6C**).

**Figure 7:**
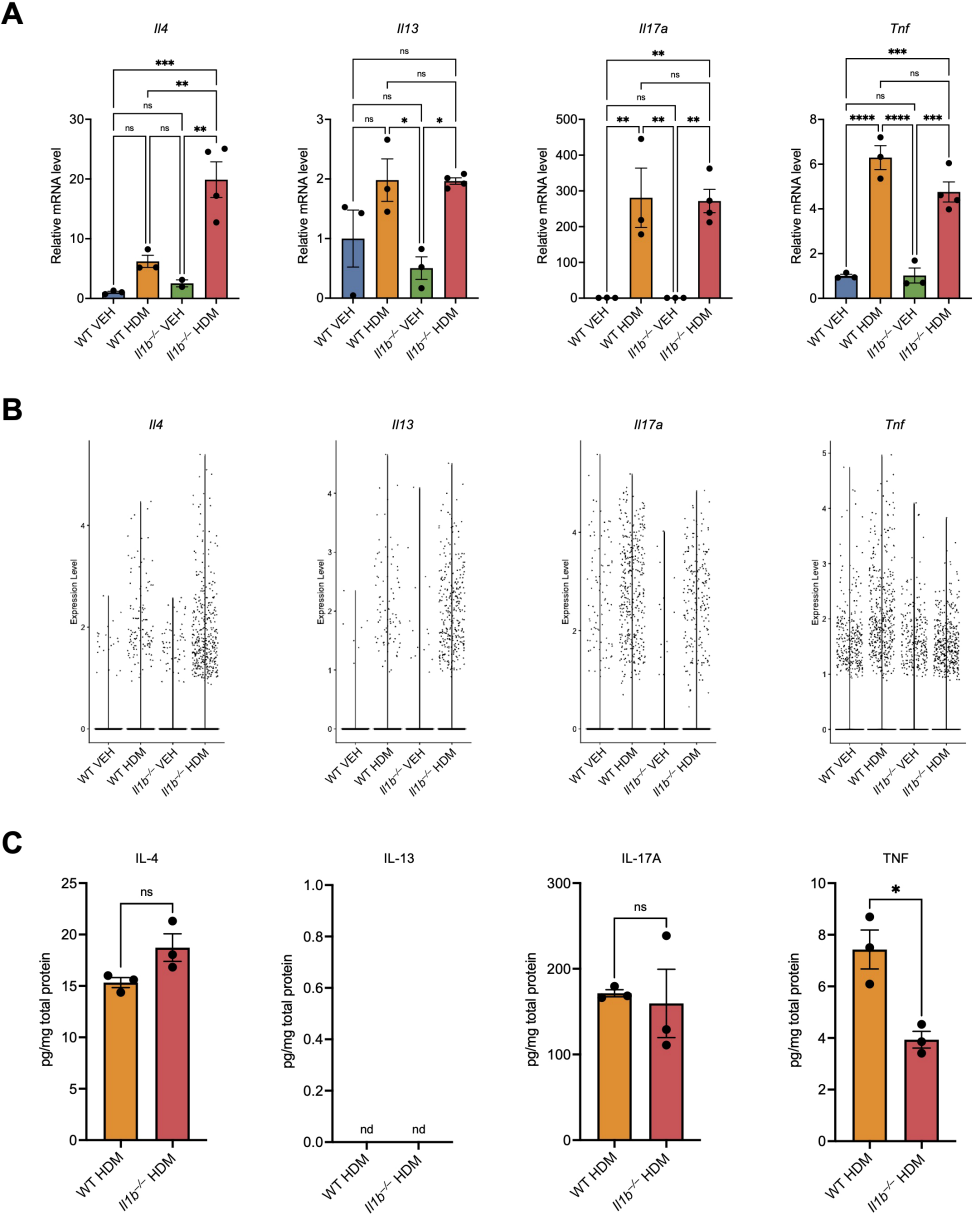
Comparative analysis of lung cytokine expression by qPCR, scRNA-seq, and ELISA. **(A)** The relative mRNA levels of the indicated cytokines were analyzed by RT-qPCR in lung homogenates of WT and *Il1b^–/–^* mice treated with VEH or HDM as shown in **Figure 1A** (n = 3–4 mice per group) and normalized to *Rplp0* (36B4) expression. The mean expression level of each cytokine in the WT VEH group was used as a reference and assigned the value of 1. **(B)** Violin plots showing the expression levels of the indicated cytokines in lung cells across the four experimental groups in the scRNA-seq dataset. Each dot represents one cell. **(C)** The protein levels of the indicated cytokines were analyzed by ELISA in lung tissue homogenates and normalized to the total amount of protein. Data are presented as mean ± SEM. Statistical significance was determined using two-sided Welch’s *t*-tests (for two-group comparisons) or one-way analysis of variance (ANOVA) followed by Bonferroni’s *post hoc* tests (for four-group comparisons). nd, not detected; ns, not significant; *p < 0.05, **p < 0.01; ***p < 0.001; ****p < 0.0001.

Next, we compared the expression of representative cytokines (IL-4, IL-13, IL-17A, and TNF) in lung tissues analyzed RT-qPCR (**Figure 7A**), scRNA-seq (**Figure 7B**), and ELISA (**Figure 7C**). Across all three techniques, the cytokine expression profiles and trends were largely consistent. Both qPCR and scRNA-seq revealed increased expression of *Il4*, *Il13*, *Il17a*, and *Tnf* in the lungs of HDM-treated WT mice compared with VEH controls (**Figures 7A, B**). In *Il1b^–/–^* HDM mice, *Il4* expression was further increased compared to WT HDM mice, consistent with the elevated proportion of CD4 Th2 cells observed in this group (**Figures 3A–C**, **Supplementary Figure 4**). *Il13* expression was similar between WT HDM and *Il1b^–/–^* HDM mice by qPCR, but the number of *Il13*-expressiong cells was increased in *Il1b^–/–^* HDM mice by scRNA-seq (**Figures 7A, B**). Likewise, *Il17a* expression was comparable between the two groups by PCR, yet the number of *Il17a*-expressiong cells was reduced in *Il1b^–/–^* HDM mice by scRNA-seq (**Figures 7A, B**). *Tnf* expression showed a decreasing trend in both qPCR and scRNA-seq analyses, consistent with the reduction in activated neutrophils and IFN neutrophils expressing *Tnf* (**Figures 4A–C**, **Supplementary Figure 5**). Although some comparisons in the ELISA analysis did not reach statistical significance, the overall patterns mirrored those observed at the transcript level, supporting concordant regulation at both mRNA and protein levels. Due to the limited tissue mass of the control groups, the lungs of WT VEH and *Il1b^–/–^* VEH mice did not yield sufficient material for both RNA and protein extraction; therefore, only HDM-treated groups were analyzed by ELISA (**Figure 7C**). Together, these results confirm the reproducibility of cytokine induction patterns across independent experiments and complementary experimental approaches, supporting the robustness of the scRNA-seq findings.

**Figure 8:**
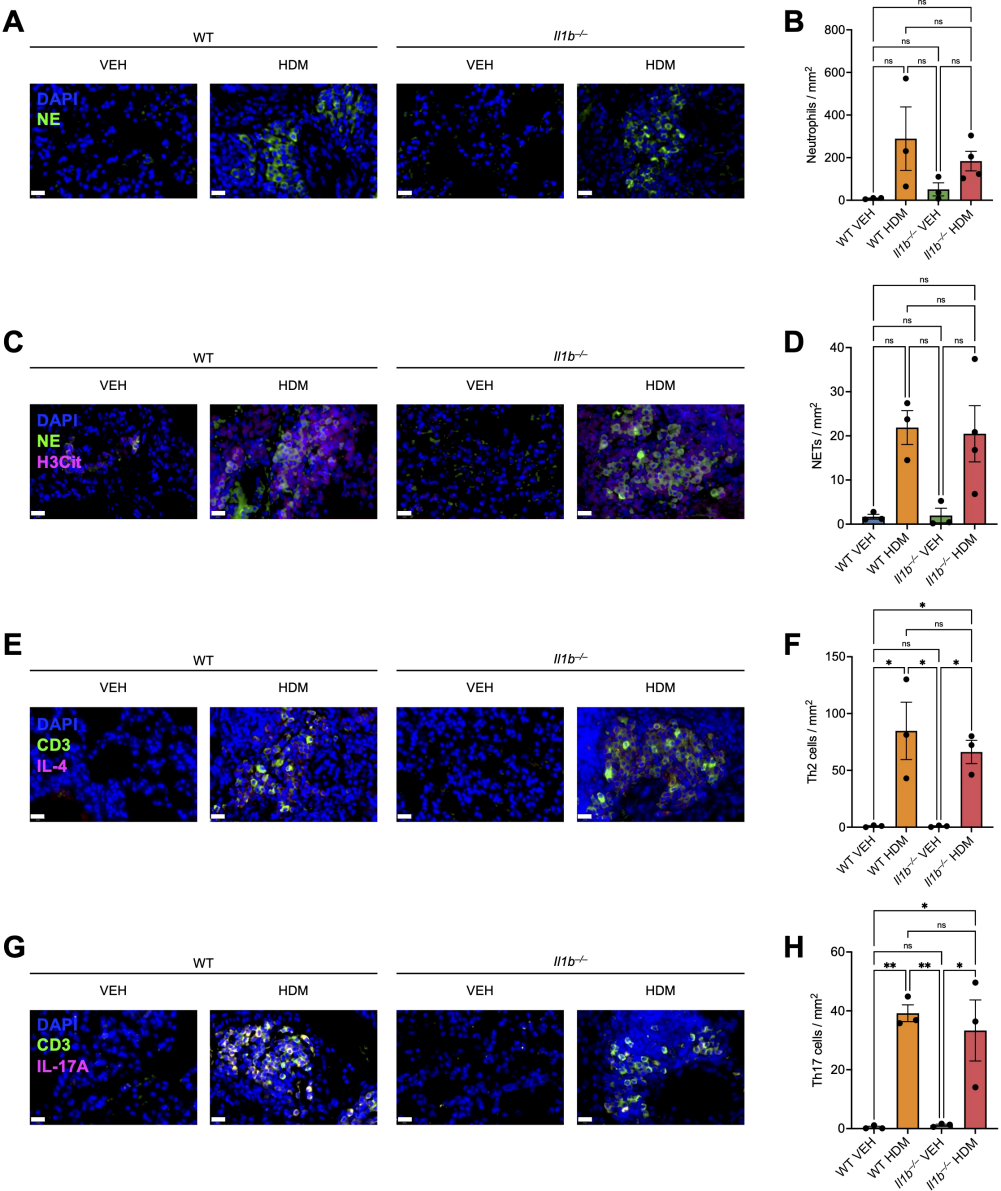
Immunofluorescence **(IF)** staining analysis of neutrophil recruitment, neutrophil extracellular trap (NET) formation, and Th2 and Th17 cell infiltration in the lungs. WT and *Il1b^–/–^* mice were treated with VEH or HDM as shown in **Figure 1A** (n = 3–4 mice per group), and lung tissue sections were processed for IF staining. **(A)** Representative images showing IF staining for neutrophil elastase (NE) on lung sections from one mouse per group. DAPI nuclear staining (blue) and NE staining (green) are shown. Scale bars, 20 μm. **(B)** Neutrophils were quantified and expressed as the number of NE+ cells per total lung area (mm^2^). **(C)** Representative images showing IF staining for NE and citrullinated histone H3 (H3Cit) on lung sections from one mouse per group. DAPI (blue), NE (green), and H3Cit (magenta). Scale bars, 20 μm. **(D)** NETs were quantified and expressed as the number of NE^+^H3Cit^+^ cells and filamentous structures per total lung area (mm^2^). **(E)** Representative images showing IF staining for CD3 and IL-4 on lung sections from one mouse per group. DAPI (blue), CD3 (green), and IL-4 (magenta). Scale bars, 20 μm. **(F)** Th2 cells were quantified and expressed as the number of CD3^+^IL-4^+^ cells per total lung area (mm^2^). **(G)** Representative images showing IF staining for CD3 and IL-17A on lung sections from one mouse per group. DAPI (blue), CD3 (green), and IL-17A (magenta). Scale bars, 20 μm. **(H)** Th17 cells were quantified and expressed as the number of CD3^+^IL-17A^+^ cells per total lung area (mm^2^). Data are presented as mean ± SEM. Statistical significance was determined using one-way analysis of variance (ANOVA) followed by Bonferroni’s *post hoc* tests. ns, not significant; *p < 0.05, **p < 0.01.

Lastly, we performed immunofluorescence (IF) staining to evaluate neutrophil recruitment, neutrophil extracellular trap (NET) formation, and Th2 and Th17 cell infiltration in the lungs across the four experimental groups. IF staining revealed pronounced neutrophil infiltration (**Figures 8A, B**) and NET formation (**Figures 8C, D**) in the lungs of both HDM-treated groups, as indicated by positive staining for neutrophil elastase (NE) alone or combined with citrullinated histone H3 (H3Cit), respectively. In contrast, minimal neutrophil and NET staining were observed in VEH-treated mice. Analysis of Th2 and Th17 cell populations showed elevated numbers of both CD3^+^IL-4^+^ (Th2) cells and CD3^+^IL-17A^+^ (Th17) cells in HDM-treated mice relative to VEH controls (**Figures 8E–H**).

**Figure 9:**
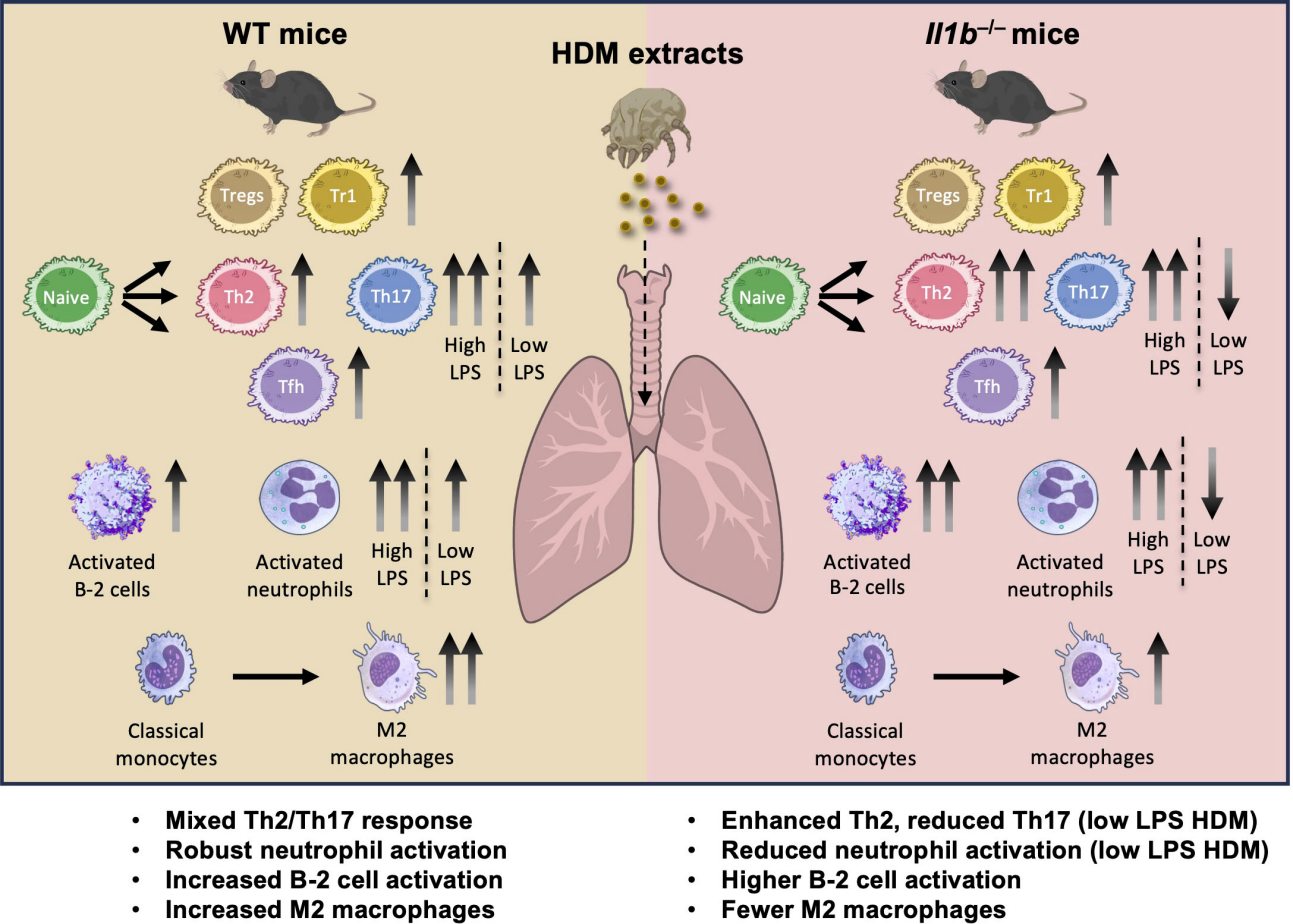
Proposed model of the effects of HDM exposure and IL-1β signaling on the lung immune microenvironment. In WT mice, chronic exposure to HDM extracts induces a mixed Th2/Th17 inflammatory response, accompanied by robust expansion of adaptive immune cells from both the B cell lineage (e.g., activated B-2 cells) and the T cell lineage (e.g., Tregs, Tr1, Th2, Th17, and T follicular helper [Tfh] cells). Several innate immune cell populations, including activated neutrophils and M2 macrophages, are also markedly increased, contributing to a complex lung microenvironment characterized with both pro-inflammatory and immunosuppressive features. In contrast, *Il1b^–/–^* mice display predominantly a Th2-skewed immune response following chronic HDM exposure, characterized by expansion of activated B-2 cells and Th2 cells, but reduced recruitment and/or differentiation of neutrophils and, to a lesser extent, M2 macrophages. However, exposure to high-LPS HDM extracts triggers strong neutrophilic and Th17 responses in both genotypes, indicating that endotoxin content can significantly influence immune polarization and override genotype-dependent effects. Together, these findings highlight the extensive immune remodeling driven by chronic HDM exposure and identify IL-1β as a central regulator of both innate and adaptive responses under low-LPS conditions. This graphical illustration was created using images from NIH BioArt source (https://bioart.niaid.nih.gov) and BioIcons (https://bioicons.com/). .

Together, these results confirm that chronic HDM exposure robustly induces neutrophil activation and Th2/Th17 cell infiltration. However, *Il1b* deficiency did not significantly alter these responses. Although the same experimental protocol was used as in the scRNA-seq study, the validation experiment employed a different batch of HDM extract containing substantially higher LPS levels. This difference likely contributed to the stronger IL-17A production, increased neutrophil recruitment, and comparable Th2/Th17 cell responses observed between WT and *Il1b^–/–^* HDM mice. A summary of the effects of chronic HDM exposure on the lung immune microenvironment, integrating scRNA-seq findings and validation experiment data, is presented in our proposed model (**Figure 9**).

## 4 Discussion

Recent advances in scRNA-seq and bioinformatics have transformed our ability to map tissue-specific immune landscapes and to explore both physiological and pathological processes within the lung (15, 16, 67). Our previous studies demonstrated that chronic HDM exposure and activation of the IL-1β signaling accelerate lung tumorigenesis in mouse models (14). In the current study, we employed scRNA-seq to comprehensively examine how chronic HDM exposure and IL-1β signaling remodel the lung microenvironment. By conducting these experiments in non-tumor-bearing mice, we specifically isolated immunological changes attributable to HDM and IL-1β, avoiding confounding effects from carcinogen-induced or oncogene-driven inflammation seen in cancer models (68, 69).

B cells represented the largest population in our dataset. Our analysis revealed a shift from resting to activated B cell states following HDM exposure. Both WT and *Il1b^–/–^* mice treated with HDM exhibited expansion of activated B-2 cells, memory B cells, GC B cells, and plasma cells, alongside reductions in naive B-2 cells, transitional B cells, and MZ B cells. These changes suggest that chronic HDM exposure delivers strong antigenic and co-stimulatory signals driving B cell clonal expansion, somatic hypermutation, and differentiation into long-lived effector cells (70). The concurrent increase in CD4^+^ FH cells supports an active CD4^+^ FH-GC B cell axis essential for robust humoral responses (71, 72). Notably, IL-1β deficiency did not impair B cell activation but appeared to enhance it, suggesting that IL-1β may serve to fine-tune the magnitude of B cell responses or that compensatory pathways in *Il1b^–/–^* mice amplify humoral immunity under chronic allergen exposure.

Our analysis revealed distinct transcription factor and signaling pathway signatures in B cell subsets from WT and *Il1b^–/–^* mice exposed to HDM. In WT mice, B cells were enriched for NF-κB, HSF1/2, TGF-β, and MAPK signaling pathways, which are canonical mediators of inflammatory and stress responses (73). In contrast, B cells in *Il1b^–/–^* mice exhibited upregulation of TNF, TRAIL, and PI3K signaling, suggesting activation of a compensatory inflammatory network in the absence of IL-1β. The enrichment of TNF and TRAIL signatures suggests that TNF superfamily cytokines may act as alternative drivers of the allergic humoral responses, consistent with known compensatory mechanisms within the IL-1β/TNF axis (74). Moreover, the strong activation of PI3K signaling, known to regulate cell proliferation, differentiation, and survival (75), correlates with the marked B cell expansion observed in *Il1b^–/–^* mice. Together, these findings indicate that in the absence of IL-1β, the immune response shifts toward a TNF-centered, pro-survival environment that sustains humoral immunity during chronic allergen exposure. In both WT and *Il1b^–/–^* mice, persistent B cell activation in response to HDM may contribute to the creation of a pro-tumor microenvironment by promoting tissue remodeling, immune suppression, and chronic inflammation.

Consistent with the established role of HDM in promoting Th2 responses, both CD4^+^ Th2 cells and ILC2s were expanded in HDM-treated mice, with an even greater expansion observed in the absence of IL-1β. Chronic HDM exposure also increased regulatory T cell populations, including Tr1 and Foxp3^+^ Tregs, via IL-1β-independent mechanisms. However, IL-1β signaling differentially shaped the CD4^+^ T cell response: its presence favored Th17 polarization, while its absence promoted a stronger Th2 response. Although IL-1β has been reported to enhance Th2 responses (76, 77), our data indicate that it is not required for Th2 cell development or function. These findings highlight divergent T cell fates in the allergic lung, which may have broader implications beyond asthma. For example, Tregs and Th17 have been implicated in shaping tissue microenvironments in chronic inflammatory diseases and cancer (78, 79). In such contexts, Tregs suppress cytotoxic T cell activity and secrete immunosuppressive cytokines like IL-10 and TGF-β, while Th17 cells drive sustained inflammation, angiogenesis, resistance to apoptosis, and IL-17-mediated neutrophil recruitment (78). Notably, inflammasome activation and IL-1β production have been shown to exacerbate allergic inflammation and promote lung tumorigenesis by enhancing Th17 differentiation (78, 79).

In contrast, the role of Th2 cells beyond allergy and parasitic infection is more nuanced and context-dependent. While Th2 cells are traditionally associated with allergic inflammation and immune responses against extracellular parasites, their signature cytokines, IL-4 and IL-13, can contribute to tumor progression by promoting M2 macrophage polarization, tissue remodeling, and enhancing the survival of malignant cells (80). Interestingly, IL-1α, rather than IL-1β, appears to play a more dominant role in HDM sensitization and Th2 immunity (81), suggesting that IL-1α may compensate in *Il1b^–/–^* mice to sustain Th2 and B cell responses.

LPS present in HDM extracts contributes to Th2 sensitization and neutrophil recruitment via TLR4 signaling (1), while IL-1β also plays a key role in promoting neutrophil infiltration in response to HDM exposure (82). Consistent with these findings, we observed a significant reduction in neutrophils and MNPs in the lungs of *Il1b^–/–^* mice, compared to HDM-treated WT controls, highlighting a critical role for IL-1β in orchestrating myeloid cell recruitment. In WT mice exposed to HDM, *Il1b* expression was detected in cMos and TRMs, but the highest expression levels were observed in neutrophil subsets, particularly IFN-stimulated and activated neutrophils. These neutrophil subsets, via IL-1β production and reactive oxygen species generation, may contribute to chronic tissue injury and the activation of tissue repair pathways. Notably, IL-1β can induce IL-6 production, a cytokine known to promote tumor progression via STAT3 activation (83). IL-1β and IL-6 can also act synergistically to promote Th17 differentiation and γδ T cell activation (84, 85). Moreover, steroid resistance in asthma has been strongly associated with neutrophilic inflammation and elevated IL-1β levels, which drive Th17 cell differentiation and IL-17A production, a pathway known to diminish steroid responsiveness (86). Together, these findings highlight the context-dependent role of IL-1β signaling in shaping lung immune responses. In our validation experiment using a different HDM extract batch with substantially higher LPS levels, we did not observe significant genotype-dependent differences in neutrophil activation or Th2/Th17 infiltration. The elevated endotoxin content likely shifted the immune response toward a more neutrophilic and Th17-skewed phenotype, reducing the differences between WT and *Il1b^–/–^* mice observed in the scRNA-seq dataset. These results underscore that batch-specific variation in HDM endotoxin content can markedly influence immune polarization and may mask or override genotype-dependent effects (87).

The lower-LPS HDM extract used for scRNA-seq elicited a more balanced Th2/Th17 response, allowing IL-1β-dependent components to be more readily resolved. Among these, TRMs, cMos, and M2 macrophages emerged as key populations modulated by chronic HDM exposure through both IL-1β-dependent and -independent mechanisms. The marked reduction in TRMs observed after HDM exposure in both WT and *Il1b^–/–^* mice is consitent with previous findings that HDM exposure induces loss of resident alveolar macrophages, the main TRM subset in the lung, followed by their replacement with long-lived monocyte-derived alveolar macrophages (88). In addition, TRMs can proliferate and polarize toward M2 macrophages upon HDM exposure (89). Given our previous observation that blocking CCL2 inhibited the lung cancer-promoting effects of HDM exposure (14), and considering that CCR2, the main CCL2 receptor, is highly expressed on monocytes and crucial for their recruitment to inflammatory sites (90), we speculate that HDM exposure triggers cMos recruitment to the lungs. These recruited cMos may differentiate into moDCs and M2 macrophages, at least partially through an IL-1β-dependent pathway.

Although basophils were identified as part of the nine major clusters in our dataset, they represented a relatively small cell population. Despite the increased *Ccl24* expression, a known chemoattractant for basophils, in M2 macrophages, the abundance of basophils was not significantly modulated by HDM exposure of IL-1β signaling and this cell type was excluded from downstream analysis.

Stromal cells, particularly endothelial cells and fibroblasts, plays critical roles in shaping a microenvironment that supports tumor growth. In our study, chronic HDM exposure markedly reduced the numbers of vascular ECs, fibroblasts, and aerocytes in both WT and *Il1b^–/–^* mice. This reduction is likely attributable due to inflammation-induced endothelial damage, vascular rarefaction, or endothelial-to-mesenchymal transition, processes commonly associated with fibrosis and tissue remodeling, as well as technical factors affecting recovery of these fragile populations. Our analysis revealed enrichment of hypoxia-related genes, heat shock proteins, and angiogenesis-associated pathways (e.g., WNT, VEGF, EGFR), supporting the presence of endothelial stress and compensatory remodeling in response to chronic HDM exposure.

Collectively, these findings highlight several cellular mechanisms through which chronic HDM exposure may promote a persistently inflamed and dysregulated immune lung microenvironment. Such an environment could facilitate immune evasion, angiogenesis, and continuous tissue remodeling, thereby creating conditions conducive to tumor initiation and progression. Further studies using tumor-bearing mice chronically exposed to HDM or other common aeroallergens will be essential to directly test this hypothesis.

### 4.1 Limitations of the study

Only male mice were used in this study to reduce biological variability due to sex-specific differences in immune cell composition and gene expression. Given the limited number of animals (n = 3–4 mice per group) and the need to pool samples for adequate cell recovery and sequencing depth, including both sexes would have introduced additional variability and confounded the interpretation of differential gene expression. Furthermore, pooling 3–4 mice per group to generate duplicate scRNA-seq libraries per condition, an approach that minimizes technical batch effects and reduces stochasticity from individual mice, precludes direct estimation of inter-mouse variability. Future studies, including male and female mice with unique libraries for each mouse, will be necessary to assess potential sex-dependent differences and inter-mouse variability in the lung microenvironment in response to HDM exposure.

Despite their well-established role in HDM-induced allergic lung inflammation, eosinophils were not detected among the annotated cell clusters. This potentially reflects both intrinsic biological properties of eosinophils and technical limitations of scRNA-seq. Mature eosinophils typically contain low levels of RNA and high concentrations of RNases and RNA-inhibitory compounds (91), making them challenging to capture in transcriptomic analyses. During quality control, their low RNA content may have led to their classification as low-quality cells, resulting in their exclusion from downstream analysis. Another potential explanation is that chronic HDM exposure may have led to the resolution of eosinophilia over time, as previously reported (7). In addition, we harvested the lung tissues 24 hours after the last HDM challenge. While this time point allowed us to capture short-lived neutrophils, peak numbers of lung eosinophils and macrophages generally occur beyond 48 hours (92). Lastly, the enzymatic dissociation of lung tissue may have led to the loss of specific cell populations that are difficult to dissociate, while also potentially damaging more fragile cell types due to excessive enzymatic stress (67). Our stringent live-cell gating strategy excluded cells expressing either Annexin V or DAPI, potentially removing pre-apoptotic or activated eosinophils. As a result, the dissociation process may not have captured the full cellular diversity present in the lung tissues. These technical limitations may partly explain the relatively low recovery of certain cell types in our analysis, including epithelial cells, endothelial cells, fibroblasts, and macrophages, resulting in overrepresentation of more easily dissociated populations such as B and T cells.

## Data Availability

The datasets presented in this study can be found in online repositories. The names of the repository/repositories and accession number(s) can be found below: GSE300531 (GEO). All analysis code used in this study is available at: https://github.com/hanbio/Mice_Lung_HDM.
