# Supplementary material for: Single-cell transcriptomics reveals immune remodeling of the murine lung microenvironment following chronic house dust mite exposure
Source: Front Immunol. 2025 Nov 27;16:1652539. doi: 10.3389/fimmu.2025.1652539 (PMC12723145; doi:10.3389/fimmu.2025.1652539)
Supplement: Supplementary file 1 [file DataSheet1.pdf]

Supplementary Material

Single-Cell Transcriptomics Reveals Immune Remodeling of the Murine Lung Microenvironment Following Chronic House Dust Mite Exposure

Han Chang[1*], Liping Zeng[1], Zahra Malakoutikhah[1], Chanond A. Nasamran[2], Scott Herdman[1], Maripat Corr[1], Kathleen M. Fisch[2], Nicholas J.G Webster[3,4], Eyal Raz[1], and Samuel Bertin[1,5*]

[1]Division of Rheumatology, Autoimmunity, and Inflammation, Department of Medicine, University of California San Diego, La Jolla, CA, USA

[2]Center for Computational Biology and Bioinformatics, School of Medicine, University of California San Diego, La Jolla, CA, USA

[3]Division of Endocrinology, Department of Medicine, University of California San Diego, La Jolla, CA, USA

[4]Medical Research Service, Veteran Affairs San Diego Healthcare System, San Diego, CA, USA

[5]Moores Cancer Center, University of California San Diego, La Jolla, CA, USA

[*]Correspondence:
Han Chang, han.chang.bio@gmail.com
Samuel Bertin, sbertin@health.ucsd.edu

This PDF file includes:

Supplementary Figures 1–11

Supplementary Tables 1-3

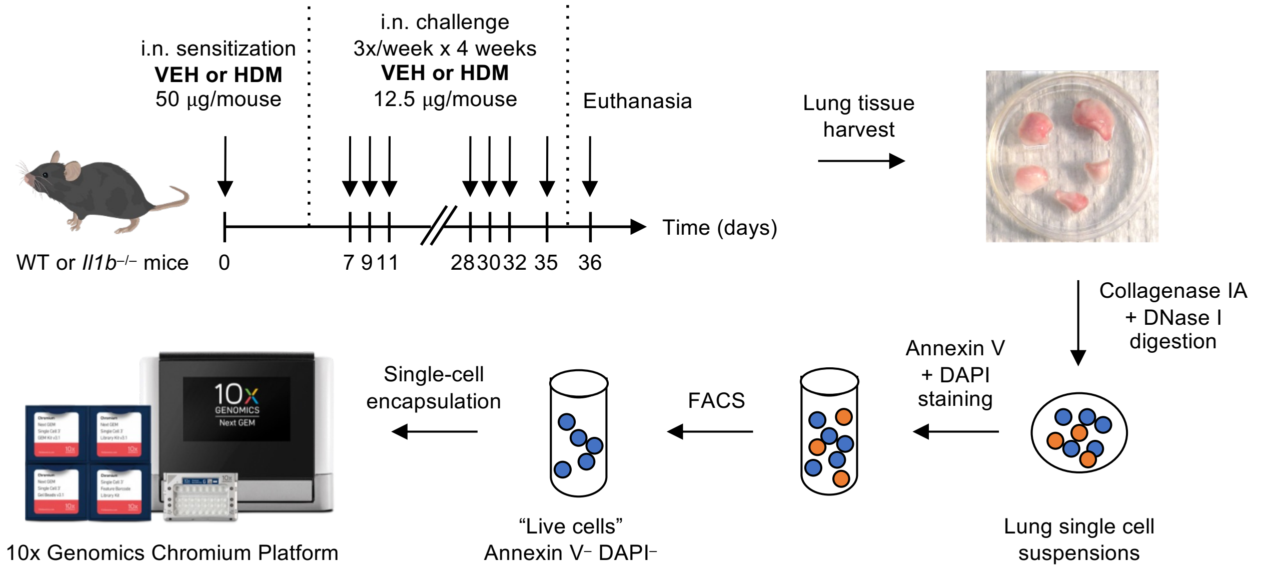

Supplementary Figure 1. Schematic overview of the study design and scRNA-seq workflow. Age- and sex-matched WT or *Il1b*$^{-/-}$ mice were treated i.n. with VEH or HDM (n = 3-4 mice per group) for 5 weeks as indicated in this schematic overview of the study design. Lung tissues from mice were harvested 24 hours after the last i.n. treatment, enzymatically digested, and the resulting single cells were stained with Annexin V and DAPI. Live (Annexin V and DAPI double-negative) single cells were sorted by FACS and approximately 16,500 cells per sample were loaded in duplicate on the 10x Genomics Chromium Controller.

A

Major Cell Types	WT VEH	WT HDM	*Il1b*$^{-/-}$ VEH	*Il1b*$^{-/-}$ HDM	Gene Markers
B cells	4,808	4,161	4,551	8,565	*Cd74, Igkc, Cd79a, Cd79b*
T cells	3,642	5,887	5,139	6,254	*Trbc2, Il7r*
NK cells	654	699	439	473	*Cd3e*$^{-}$*, Ncr1, Klrb1c, Gzma*
Neutrophils	672	940	625	143	*S100a9, S100a8, Cxcr2, Csf3r*
MNPs	411	351	612	171	*Adgre4, Lyz2*
Endothelial cells	856	86	564	7	*Cdh5, Cldn5, Vwf*
Fibroblasts	128	7	142	0	*Sparc, Col3a1*
Basophils	29	28	13	49	*Mcpt8, Cd63*
Epithelial cells	9	10	2	12	*Sftpa1, Sftpb, Sftpd, Scgb1a1*

B

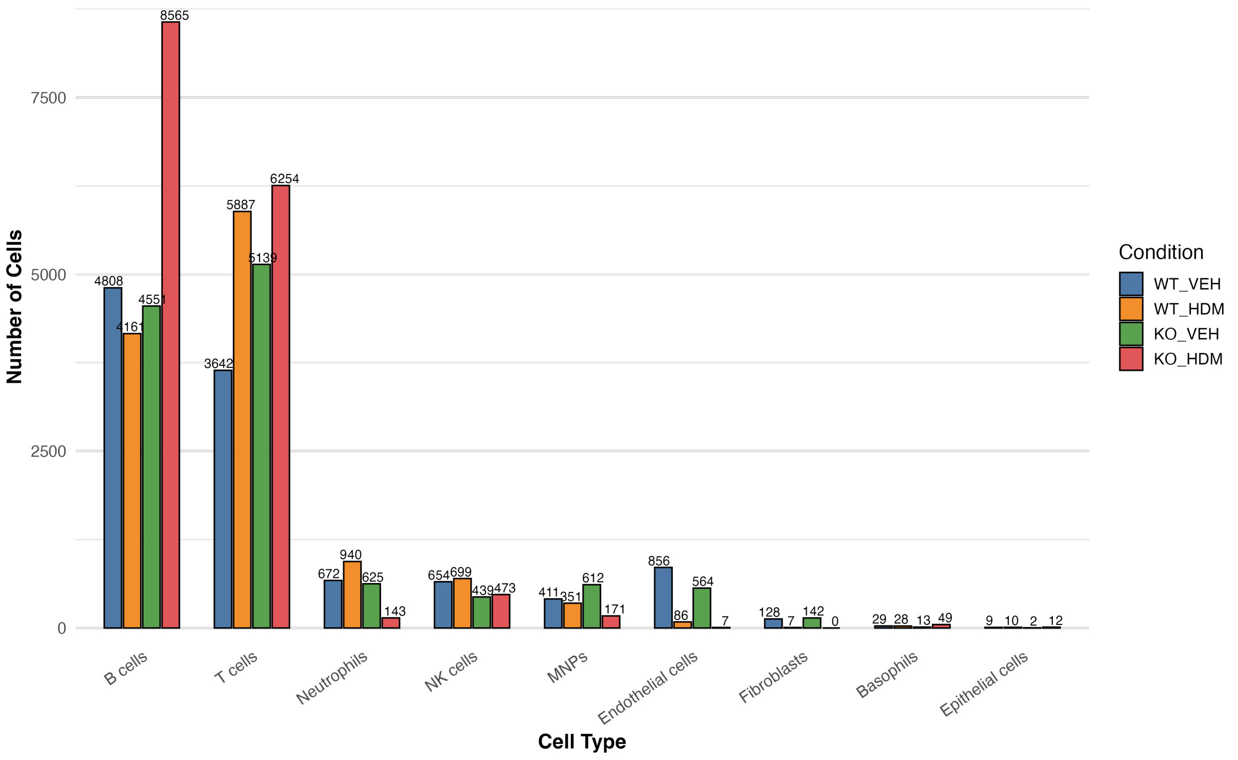

Supplementary Figure 2. Final major clusters. A) Table presenting the nine major cell clusters identified across the four experimental conditions, along with corresponding cell counts per group. Subclustering was performed to enhance resolution, followed by reintegration into the initial clustering framework to refine cell type annotations and remove technical artifacts (e.g., doublets, ambient RNA). Each cluster exhibits distinct expression of canonical marker genes consistent with known cell identities. **B)** Histogram showing absolute cell numbers of each cell cluster across the four experimental groups, corresponding to the values shown in panel A, illustrating the distribution and abundance of cell subsets identified by single-cell analysis.

A

Cell Subtypes	WT VEH	WT HDM	*Il1b*$^{-/-}$ VEH	*Il1b*$^{-/-}$ HDM	Cell Type Markers
Naive B-2 cells	4266	2080	3866	2068	*Klf2, Ccr7, Ighd, Ighm*
Activated B-2 cells	108	1396	111	5451	*Cr2, Samsn1, Ifi30, Ciita*
Memory B cells	23	156	17	402	*Ighg1, Igha, Apoe, Ccr6, Cd80*
Transitional B cells	233	162	117	66	*Iglc1, Vpreb3, Cd24a*
MZ B cells	77	29	285	133	*Plac8, Mzb1, Ighd, Ighm*
IFN B cells	92	122	139	167	*Stat1, Ifi27l2a, Ifit3, Isg15*
GC B cells	5	121	5	203	*Mybl1, Aicda, Mki67, Pclaf*
Plasma cells	4	95	11	75	*Jchain, Xbp1, Igha, Ighg1, Slpi*

B

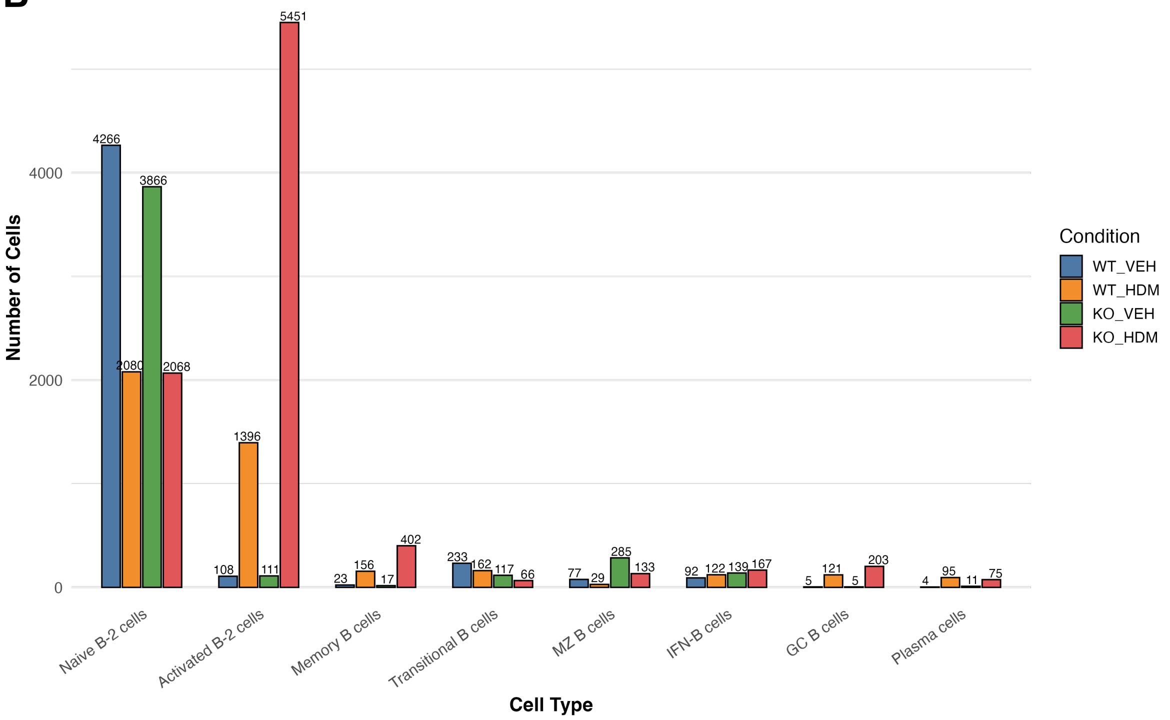

Supplementary Figure 3. B cell subclustering results. A) Table presenting the number of cells in each B subcluster across the four experimental conditions. The canonical and supportive marker genes listed were used to annotate the following B cell subtypes: naive B-2 cells, activated B-2 cells, memory B cells, transitional B cells, marginal zone B cells (MZ B cells), interferon-activated B cells (IFN B

cells), germinal center B cells (GC B cells), and plasma cells. **B)** Histogram showing absolute cell numbers of each B cell subcluster across the four experimental groups, corresponding to the values shown in panel A, illustrating the distribution and abundance of B cell subsets identified by single-cell analysis.

A

Cell Subtypes	WT VEH	WT HDM	*Il1b*$^{-/-}$ VEH	*Il1b*$^{-/-}$ HDM	Cell Markers
CD4 naive	1212	1009	1881	1126	*Cd4, Ccr7, Sell, Lef1, Tcf7, Il7r*
CD8 naive	983	994	1591	1111	*Cd8a, Cd8b1, Ccr7, Sell, Lef1, Tcf7, Il7r*
NK cells	654	699	439	473	*Cd3e*$^{-}$, *Ncr1, Klrb1c, Gzma, Klrk1*
CD4 EM	481	972	365	337	*Cd4, Il21, Cd44, Tcf, Maf, Rora, Cd40lg*
CD8 cytotoxic	284	410	454	637	*Cd8a, Cd8b1, Gzmb, Gzmk, Ccl5, Nkg7*
Tr1	54	806	30	886	*Cd4, Maf, Rbpj, Ahr, Foxp3*$^{-}$, *Gata3*^{low}, *Il4*$^{-}$
CD8 CM	294	453	190	422	*Cd8a/b1, Sell, Lef1, Tcf7, Il7r, Eomes, Il2rb, Bcl2, Ccl5, Nkg7, Ly6c2*
Foxp3^{+} Tregs	61	386	54	355	*Cd4, Foxp3, Ikzf2, Il2ra, Tnfrsf4, Itgb8*
CD4 Th2	1	132	106	523	*Cd4, Maf, Il1rl1, Gata3, Il4, Il13, Pparg*
CD4 FH	7	227	7	275	*Cd4, Tcf7, Lrig1, Maf, Tox2, Il21, Cd40lg*
NKT cells	46	155	141	157	*Cd3e, Itga1, Xcl1, Zbtb16, Klrb1b, Tyrobp, Klrk, Trdc-*
γδ T cells	93	92	93	141	*Cd3e, Cd4*$^{-}$, *Cd8a*$^{-}$*/b1*$^{-}$, *Trdc, Tcrgc1, Zbtb16, Il23r, Il1r1, Il7r, Maf, Rora*
IFN T cells	35	138	81	101	*Cd3e, Ifit1, Isg15, Rsad2*
Treg naive	61	40	83	26	*Foxp3, Sell, Ccr7, Il2ra, Lrig1*
ILC2s	17	29	35	120	*Cd3e-, Rora, Il1rl1, Arg1, Il7r, Gata3*
Prolif T cells	13	44	28	37	*Mki67, Birc5, Cenpf, Top2a, Cdk1*

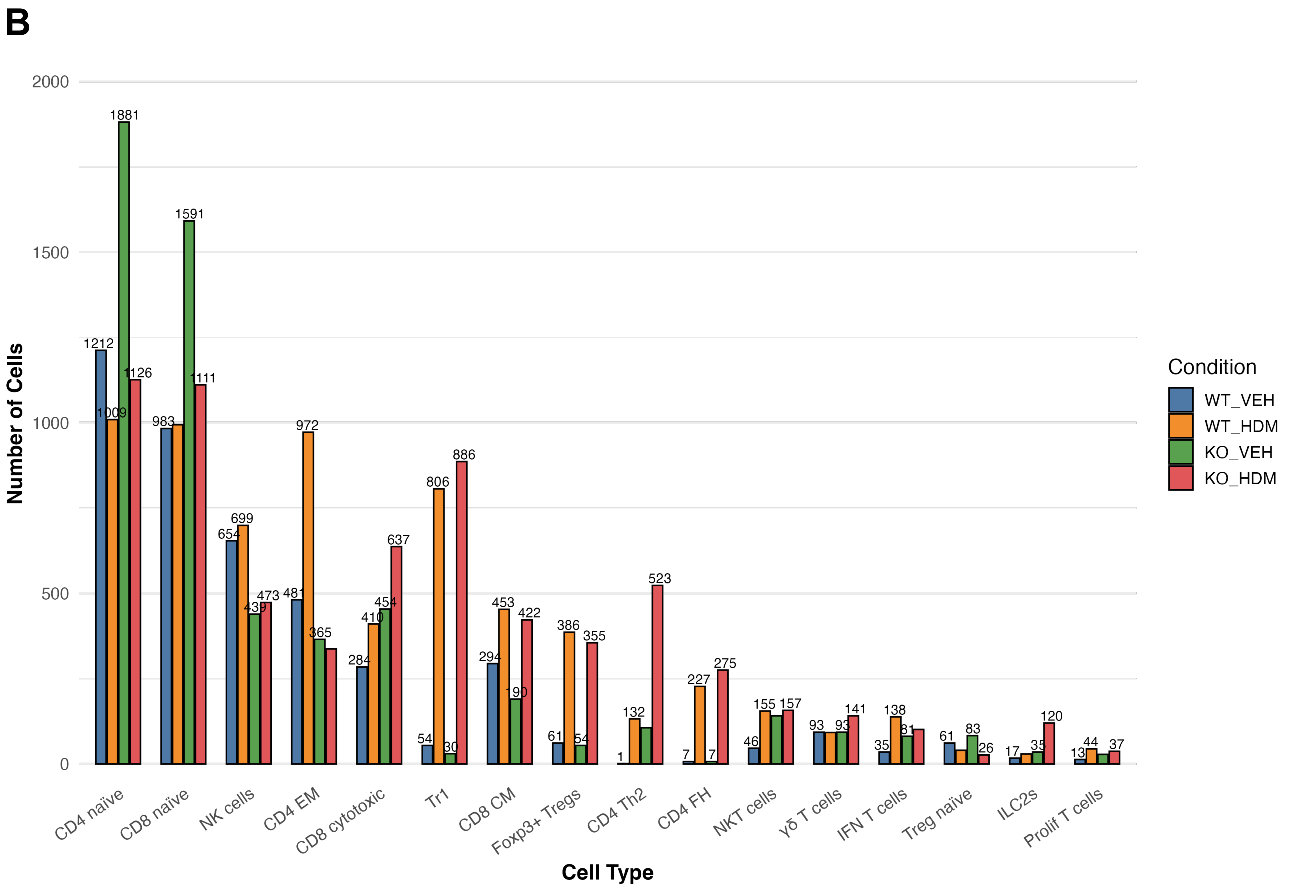

Supplementary Figure 4. T cell subclustering results. A) Table presenting the number of cells in each T cell subcluster across the four experimental conditions. The canonical marker and supportive marker genes listed were used to annotate the following T cell subtypes: naive CD4 T cells (CD4 naive), naive CD8 T cells (CD8 naive), natural killer cells (NK cells), effector memory CD4 T cells (CD4 EM), cytotoxic CD8 T cells (CD8 cytotoxic), type 1 regulatory T cells (Tr1), central memory CD8 T cells (CD8 CM), CD4 Foxp3$^+$ regulatory T cells (*Foxp3*$^+$ Treg), CD4 T helper 2 cells (CD4 Th2), follicular helper CD4 T cells (CD4 FH), natural killer T cells (NKT cells), γδ T cells, T cells with high interferon-stimulated gene expression (IFN T cells), naive regulatory T cells (Treg naive), type 2 innate lymphoid cells (ILC2s), and T cells expressing proliferation-related genes (Prolif T cells). **B)** Histogram showing absolute cell numbers of each T cell subcluster across the four experimental groups, corresponding to the values shown in panel A, illustrating the distribution and abundance of T cell subsets identified by single-cell analysis.

A

Cell Subtypes	WT VEH	WT HDM	*Il1b*$^{-/-}$ VEH	*Il1b*$^{-/-}$ HDM	Gene Markers
Mature neutrophils	298	307	339	88	*S100a8, S100a9, Retnlg, Csf3r, Lcn2*
Resting neutrophils	303	194	256	27	*Clec4d, Slc7a11, Nlrp3, C5ar1*
Activated neutrophils	56	373	19	25	*Il1a, Tnf, Ccl3, Clec5a, Il1rn*
IFN neutrophils	15	66	11	3	*Ifit1, Ifi204, Rsad2, Isg15, Cxcl10*

B

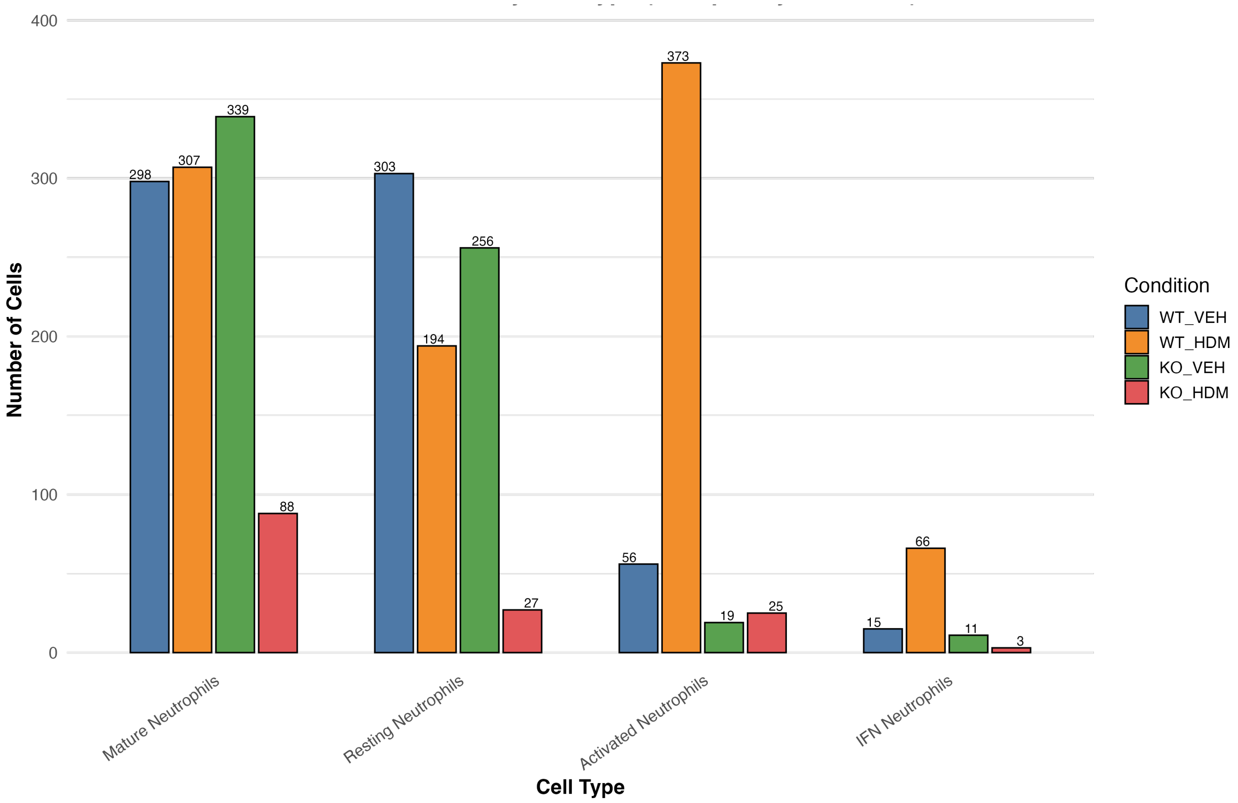

Supplementary Figure 5. Neutrophil subclustering results. A) Table presenting the cell numbers for each neutrophil subcluster across the four experimental conditions. Four neutrophil subtypes were identified: mature neutrophils, resting neutrophils, activated neutrophils, and neutrophils with high interferon-stimulated gene expression (IFN neutrophils). **B)** Histogram showing absolute cell numbers of each neutrophil subcluster across the four experimental groups, corresponding to the values shown in panel A, illustrating the distribution and abundance of neutrophil subsets identified by single-cell analysis.

A

Subcluster	WT VEH	WT HDM	*Il1b*$^{-/-}$ VEH	*Il1b*$^{-/-}$ HDM	Gene Markers
Vascular ECs	771	76	530	6	*Pecam, Cldn5, Ptprb, Tek*
TRMs	166	97	360	40	*Lyz2, Adgre4, Pparg, Clec4a1, Cd300e*
cMos	191	122	200	46	*Lyz2, Ccr2, Fn1, F13a1, Mafb, Csf1r*
moDCs	29	56	31	39	*Cd209a, Batf3, Cxcl16, Aif1*
Resting fibroblasts	73	4	62	0	*Dcn, Lum, Mgp, Hhip, Igfbp5*
Activated fibroblasts	55	3	80	0	*Postn, Trpc6, Adcy8*
Aerocytes	85	10	34	1	*Ednrb, Emp2, Car4,*
M2 macrophages	9	67	9	33	*Chil3, Arg1, Igf1, Mrc1, Fabp4*
pDCs	16	9	12	13	*Siglech, Tcf4, Bst2, Irf8, Grm8*
AT2 cells	9	10	2	12	*Sftpa1, Sftpb, Sftpd, Cbr2, Scgb1a1*

B

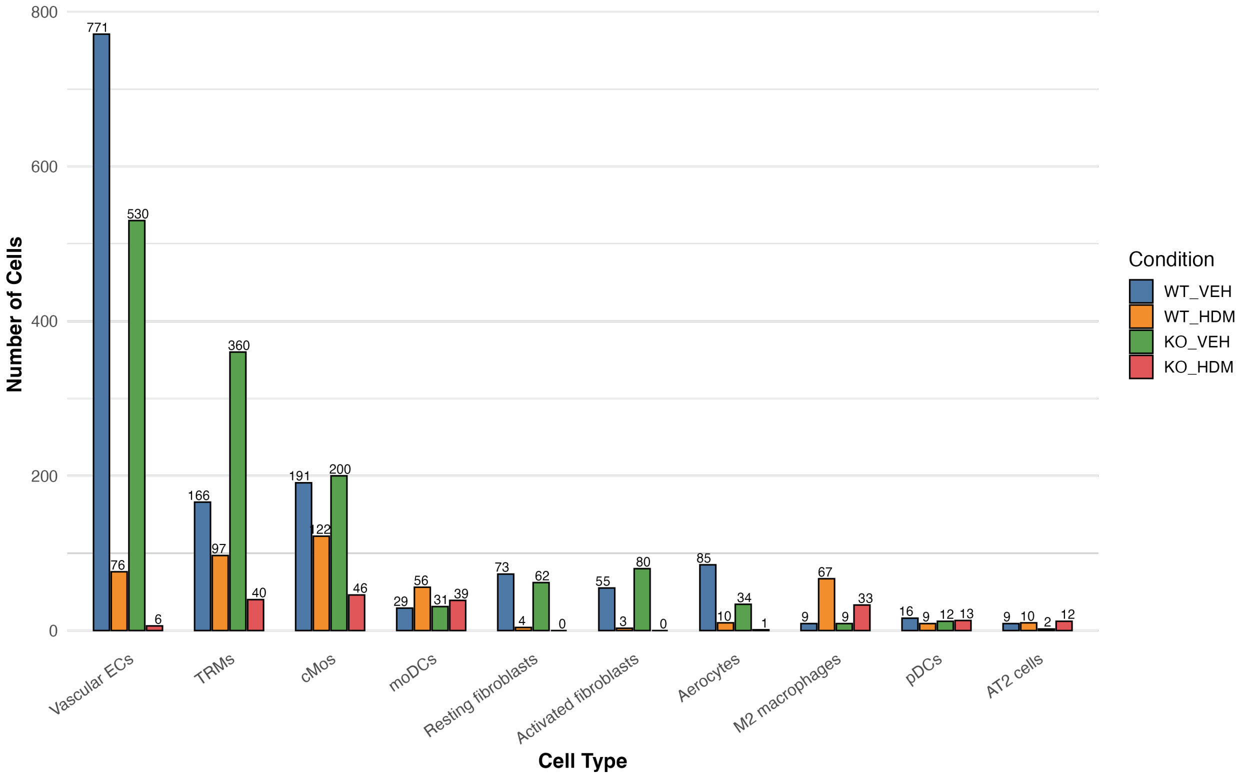

Supplementary Figure 6. MNP and stromal cell subclustering results. A) Table presenting the cell numbers for each mononuclear phagocytes (MNPs) and stromal cell subtypes across the four experimental conditions. After merging and subclustering of MNPs and stromal cells, canonical and supportive marker genes in the table were used to annotate the following subtypes: vascular endothelial cells (Vascular ECs), tissue-resident macrophages (TRMs), classical monocytes (cMos), monocyte-derived dendritic cells (moDCs), resting fibroblasts, activated fibroblasts, aerocytes, alternatively activated macrophages (M2 macrophages), plasmacytoid dendritic cells (pDCs), and alveolar type 2 epithelial cells (AT2 cells). **B)** Histogram showing absolute cell numbers of each MNP and stromal cell subcluster across the four experimental groups, corresponding to the values shown in panel A, illustrating the distribution and abundance of MNP and stromal cell subsets identified by single-cell analysis.

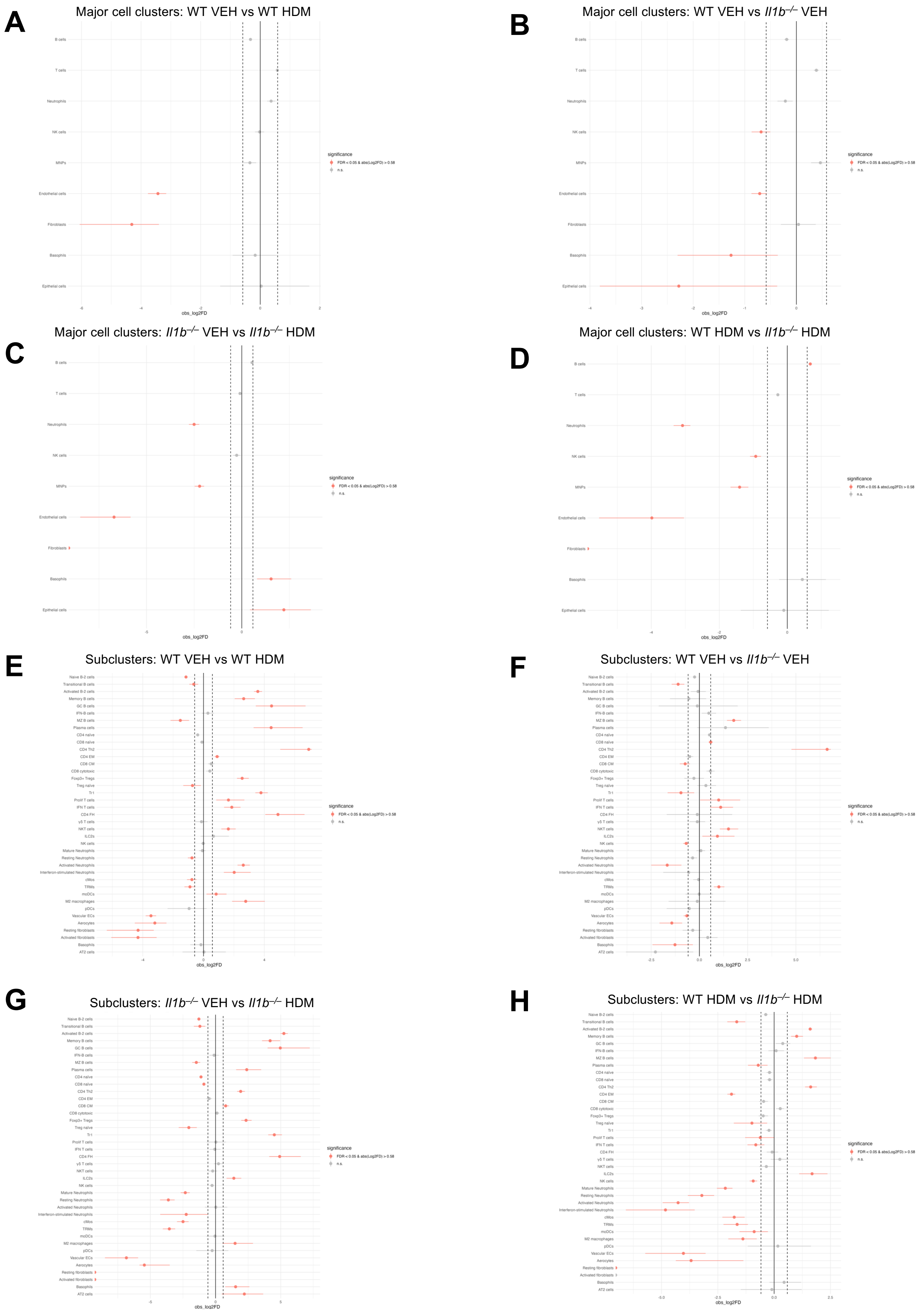
A
Major cell clusters: WT VEH vs WT HDM
B
Major cell clusters: WT VEH vs Il1b–/– VEH
C
Major cell clusters: Il1b–/– VEH vs Il1b–/– HDM
D
Major cell clusters: WT HDM vs Il1b–/– HDM
E
Subclusters: WT VEH vs WT HDM
F
Subclusters: WT VEH vs Il1b–/– VEH
G
Subclusters: Il1b–/– VEH vs Il1b–/– HDM
H
Subclusters: WT HDM vs Il1b–/– HDM
B cells
T cells
Neutrophils
NK cells
MNPs
Endothelial cells
Fibroblasts
Basophils
Epithelial cells
obs_log2FD
significance
FDR < 0.05 & abs(Log2FD) > 0.58
n.s.

Supplementary Figure 7. Statistical analyses for cell type population changes across experimental groups. Permutation test results in the four experimental conditions for **(A-D)** the nine major cell clusters and **(E-H)** the four subclusters. Red clusters indicate significant enrichment between the two groups (FDR <0.05 and $|\log_2$ fold enrichment$|$ >0.58), while grey clusters are not significant (n.s.).

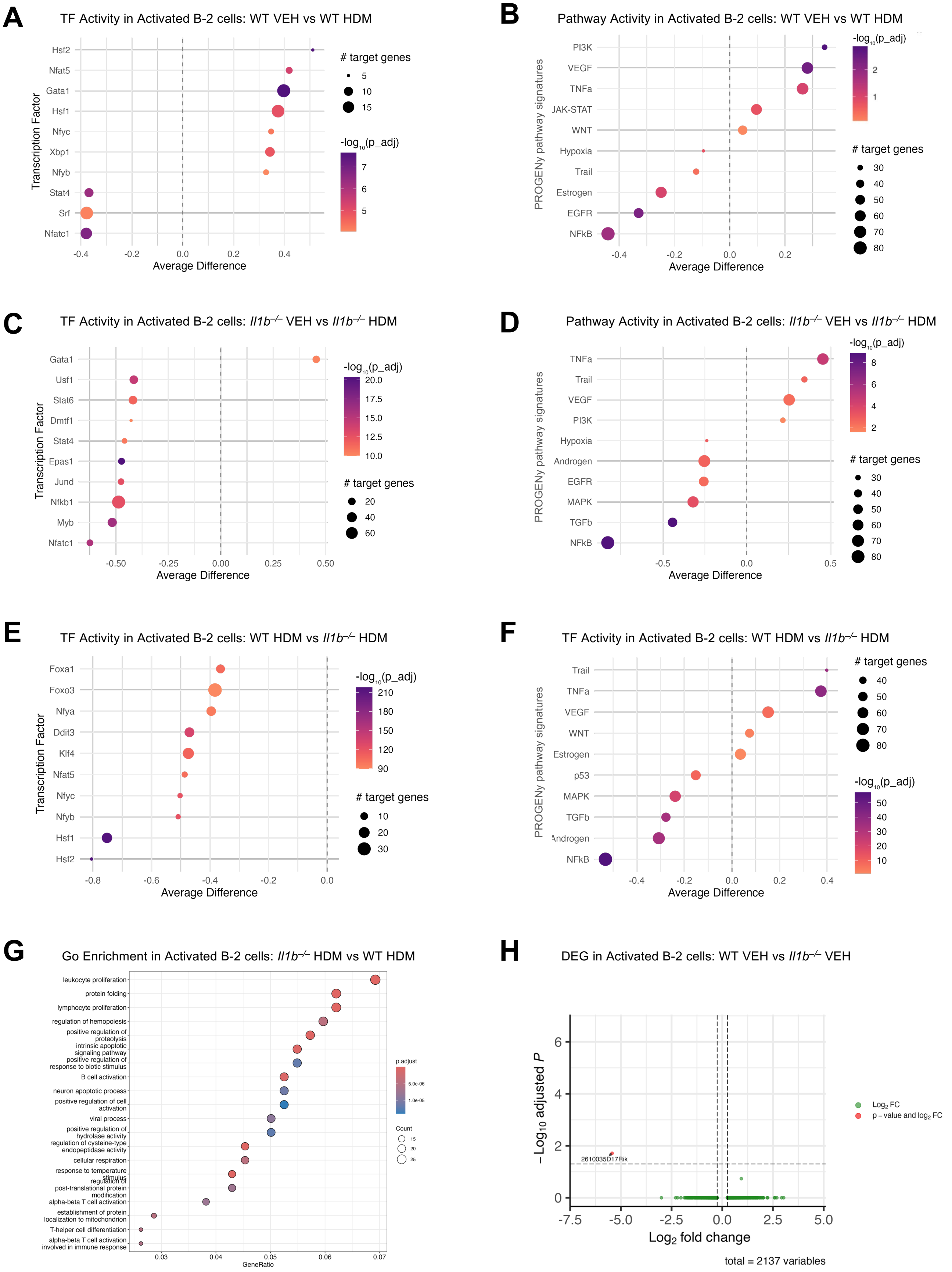
A
TF Activity in Activated B-2 cells: WT VEH vs WT HDM
Transcription Factor
Hsf2
Nfat5
Gata1
Hsf1
Nfyc
Xbp1
Nfyb
Stat4
Srf
Nfatc1
-0.4
-0.2
0.0
0.2
0.4
Average Difference
target genes
5
10
15
-log10(p_adj)
7
6
5
B
Pathway Activity in Activated B-2 cells: WT VEH vs WT HDM
PROGENy pathway signatures
PI3K
VEGF
TNFa
JAK-STAT
WNT
Hypoxia
Trail
Estrogen
EGFR
NFkB
-0.4
-0.2
0.0
0.2
Average Difference
-log10(p_adj)
2
1
target genes
30
40
50
60
70
80
C
TF Activity in Activated B-2 cells: Il1b−/− VEH vs Il1b−/− HDM
Transcription Factor
Gata1
Usf1
Stat6
Dmtf1
Stat4
Epas1
Jund
Nfkb1
Myb
Nfatc1
-0.50
-0.25
0.00
0.25
0.50
Average Difference
-log10(p_adj)
20.0
17.5
15.0
12.5
10.0
target genes
20
40
60
D
Pathway Activity in Activated B-2 cells: Il1b−/− VEH vs Il1b−/− HDM
PROGENy pathway signatures
TNFa
Trail
VEGF
PI3K
Hypoxia
Androgen
EGFR
MAPK
TGFb
NFkB
-0.5
0.0
0.5
Average Difference
-log10(p_adj)
8
6
4
2
target genes
30
40
50
60
70
80
E
TF Activity in Activated B-2 cells: WT HDM vs Il1b−/− HDM
Transcription Factor
Foxa1
Foxo3
Nfya
Ddit3
Klf4
Nfat5
Nfyc
Nfyb
Hsf1
Hsf2
-0.8
-0.6
-0.4
-0.2
0.0
Average Difference
-log10(p_adj)
210
180
150
120
90
target genes
10
20
30
F
TF Activity in Activated B-2 cells: WT HDM vs Il1b−/− HDM
PROGENy pathway signatures
Trail
TNFa
VEGF
WNT
Estrogen
p53
MAPK
TGFb
Androgen
NFkB
-0.4
-0.2
0.0
0.2
0.4
Average Difference
target genes
40
50
60
70
80
-log10(p_adj)
50
40
30
20
10
G
Go Enrichment in Activated B-2 cells: Il1b−/− HDM vs WT HDM
leukocyte proliferation
protein folding
lymphocyte proliferation
regulation of hemopoiesis
positive regulation of proteolysis
intrinsic apoptotic signaling pathway
positive regulation of response to biotic stimulus
B cell activation
neuron apoptotic process
positive regulation of cell activation
viral process
positive regulation of hydrolase activity
regulation of cysteine-type endopeptidase activity
cellular respiration
response to temperature stimulus
regulation of post-translational protein modification
alpha-beta T cell activation
establishment of protein localization to mitochondrion
T-helper cell differentiation
alpha-beta T cell activation involved in immune response
0.03
0.04
0.05
0.06
0.07
GeneRatio
p.adjust
5.0e-06
1.0e-05
Count
15
20
25
H
DEG in Activated B-2 cells: WT VEH vs Il1b−/− VEH
– Log10 adjusted P
6
4
2
0
2610035D17Rik
-7.5
-5.0
-2.5
0.0
2.5
5.0
Log2 fold change
Log2 FC
p – value and log2 FC
total = 2137 variables

Supplementary Figure 8. Transcription Factor (TF) analysis, pathway enrichment, Gene Ontology (GO) analysis, and differential gene expression analysis for activated B-2 Cells. A) TF activity in activated B-2 cells from WT VEH and WT HDM mice. **B)** Pathway activity in activated B-2 cells from WT VEH and WT HDM mice. **C)** TF activity in activated B-2 cells from *Il1b*$^{-/-}$ VEH and *Il1b*$^{-/-}$ HDM mice. **D)** Pathway activity in activated B-2 cells from *Il1b*$^{-/-}$ VEH and *Il1b*$^{-/-}$ HDM mice. **E)** TF activity in activated B-2 cells from WT HDM and *Il1b*$^{-/-}$ HDM mice. **F)** Pathway activity in activated B-2 cells from WT HDM and *Il1b*$^{-/-}$ HDM mice. **G)** GO enrichment analysis in activated B-2 cells from *Il1b*$^{-/-}$ HDM and WT HDM mice. **H)** DEG analysis in activated B-2 cells from WT VEH and *Il1b*$^{-/-}$ VEH mice. Circle size indicates the proportion of genes associated with each term that are represented in the dataset, while color denotes statistical significance based on the adjusted p-value.

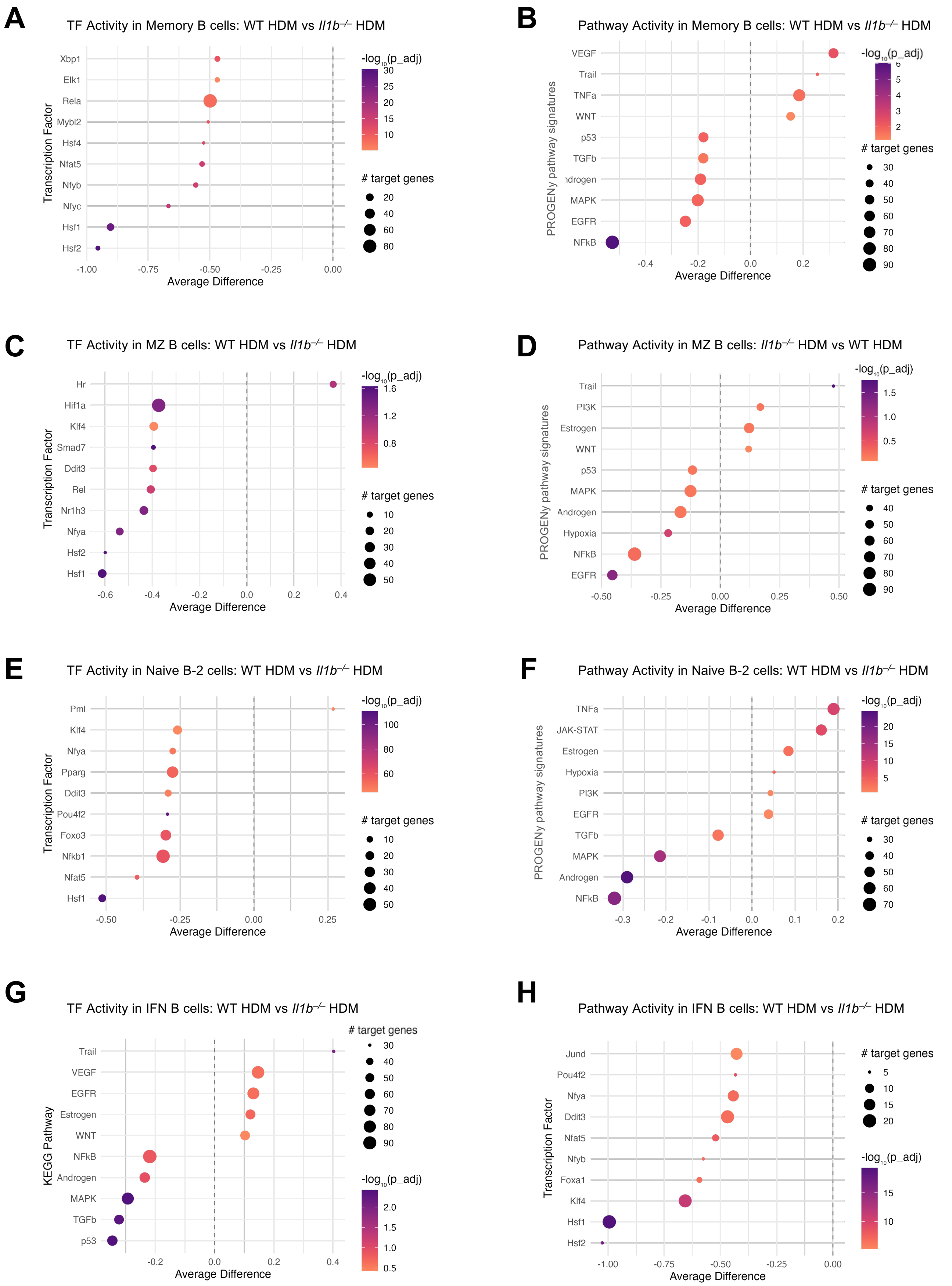
A
TF Activity in Memory B cells: WT HDM vs Il1b−/− HDM
Transcription Factor
Xbp1
Elk1
Rela
Mybl2
Hsf4
Nfat5
Nfyb
Nfyc
Hsf1
Hsf2
-1.00
-0.75
-0.50
-0.25
0.00
Average Difference
-log10(p_adj)
30
25
20
15
10
target genes
20
40
60
80
B
Pathway Activity in Memory B cells: WT HDM vs Il1b−/− HDM
PROGENy pathway signatures
VEGF
Trail
TNFa
WNT
p53
TGFb
ndrogen
MAPK
EGFR
NFkB
-0.4
-0.2
0.0
0.2
Average Difference
-log10(p_adj)
6
5
4
3
2
target genes
30
40
50
60
70
80
90
C
TF Activity in MZ B cells: WT HDM vs Il1b−/− HDM
Transcription Factor
Hr
Hif1a
Klf4
Smad7
Ddit3
Rel
Nr1h3
Nfya
Hsf2
Hsf1
-0.6
-0.4
-0.2
0.0
0.2
0.4
Average Difference
-log10(p_adj)
1.6
1.2
0.8
target genes
10
20
30
40
50
D
Pathway Activity in MZ B cells: Il1b−/− HDM vs WT HDM
PROGENy pathway signatures
Trail
PI3K
Estrogen
WNT
p53
MAPK
Androgen
Hypoxia
NFkB
EGFR
-0.50
-0.25
0.00
0.25
0.50
Average Difference
-log10(p_adj)
1.5
1.0
0.5
target genes
40
50
60
70
80
90
E
TF Activity in Naive B-2 cells: WT HDM vs Il1b−/− HDM
Transcription Factor
Pml
Klf4
Nfya
Pparg
Ddit3
Pou4f2
Foxo3
Nfkb1
Nfat5
Hsf1
-0.50
-0.25
0.00
0.25
Average Difference
-log10(p_adj)
100
80
60
target genes
10
20
30
40
50
F
Pathway Activity in Naive B-2 cells: WT HDM vs Il1b−/− HDM
PROGENy pathway signatures
TNFa
JAK-STAT
Estrogen
Hypoxia
PI3K
EGFR
TGFb
MAPK
Androgen
NFkB
-0.3
-0.2
-0.1
0.0
0.1
0.2
Average Difference
-log10(p_adj)
20
15
10
5
target genes
30
40
50
60
70
G
TF Activity in IFN B cells: WT HDM vs Il1b−/− HDM
KEGG Pathway
Trail
VEGF
EGFR
Estrogen
WNT
NFkB
Androgen
MAPK
TGFb
p53
-0.2
0.0
0.2
0.4
Average Difference
target genes
30
40
50
60
70
80
90
-log10(p_adj)
2.0
1.5
1.0
0.5
H
Pathway Activity in IFN B cells: WT HDM vs Il1b−/− HDM
Transcription Factor
Jund
Pou4f2
Nfya
Ddit3
Nfat5
Nfyb
Foxa1
Klf4
Hsf1
Hsf2
-1.00
-0.75
-0.50
-0.25
0.00
Average Difference
target genes
5
10
15
20
-log10(p_adj)
15
10

Supplementary Figure 9. Enrichment analysis of TFs and pathways associated with DEGs in other B cell subtypes. A) TF activity in memory B cells from WT HDM and *Il1b*$^{-/-}$ HDM mice. **B)** Pathway activity in memory B cells from WT HDM and *Il1b*$^{-/-}$ HDM mice. **C)** TF activity in MZ B cells from WT HDM and *Il1b*$^{-/-}$ HDM mice. **D)** Pathway activity in MZ B cells from WT HDM and *Il1b*$^{-/-}$ HDM mice. **E)** TF activity in naive B-2 cells from WT HDM and *Il1b*$^{-/-}$ HDM mice. **F)** Pathway activity in naive B-2 cells from WT HDM and *Il1b*$^{-/-}$ HDM mice. **G)** TF activity in IFN B cells from WT HDM and *Il1b*$^{-/-}$ HDM mice. **H)** Pathway activity IFN B cells from WT HDM and *Il1b*$^{-/-}$ HDM mice. Circle size indicates the proportion of genes associated with each term that are represented in the dataset, while color denotes statistical significance based on the adjusted p-value.

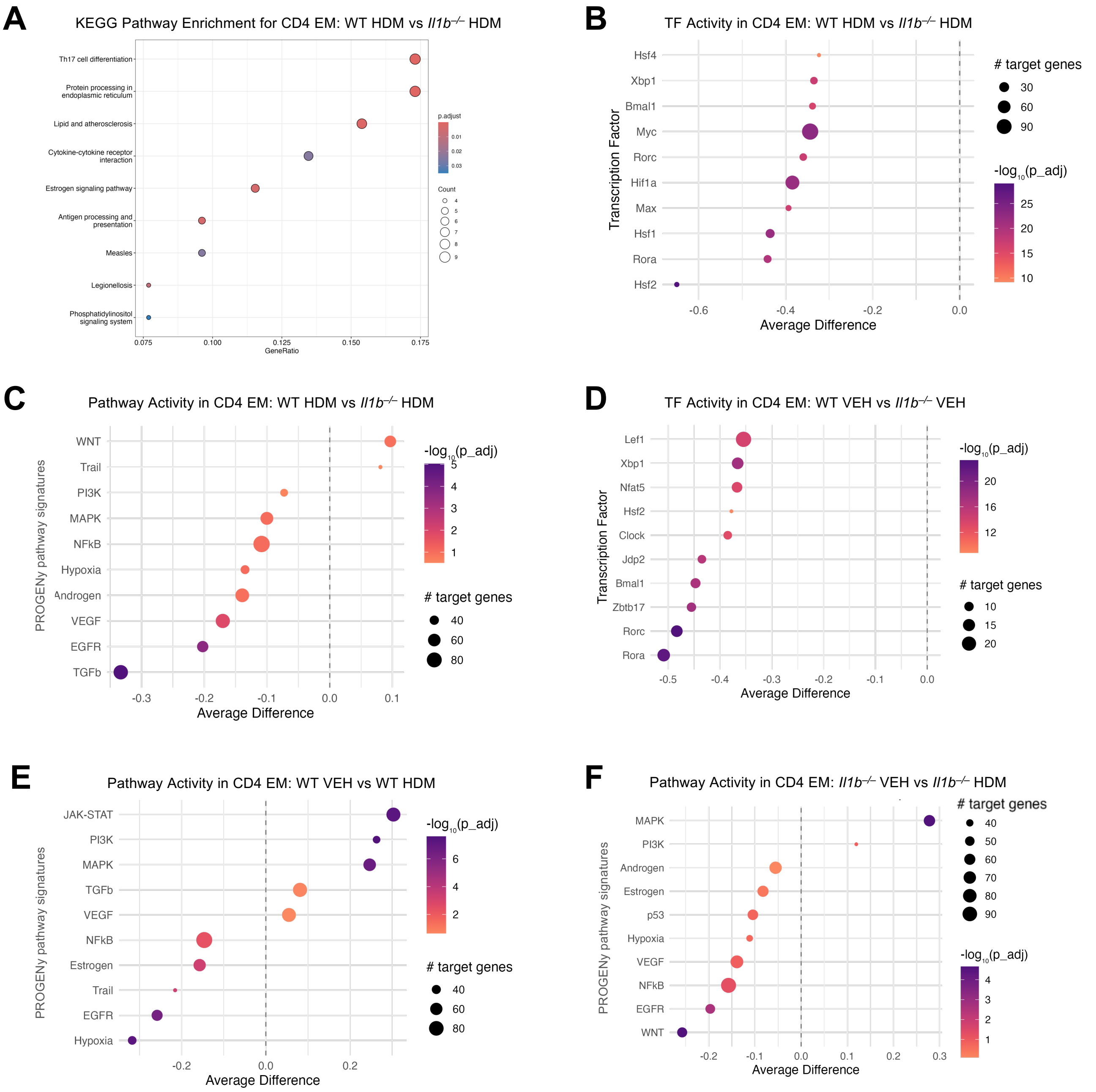

Supplementary Figure 10. Enrichment analysis of TFs and pathways associated with DEGs in CD4 EM. A) KEGG pathway enrichment TF activity in CD4 EM from *Il1b*$^{-/-}$ HDM and WT HDM mice. **B)** TF activity in CD4 EM from WT HDM and *Il1b*$^{-/-}$ HDM mice. **C)** Pathway activity in CD4 EM from WT HDM and *Il1b*$^{-/-}$ HDM mice. **D)** TF activity in CD4 EM from WT VEH and *Il1b*$^{-/-}$ VEH mice. **E)** Pathway activity in CD4 EM from WT VEH and WT HDM mice. **F)** TF activity in CD4 EM from *Il1b*$^{-/-}$ VEH and *Il1b*$^{-/-}$ HDM mice. Circle size indicates the proportion of genes associated with each term that are represented in the dataset, while color denotes statistical significance based on the adjusted p-value.

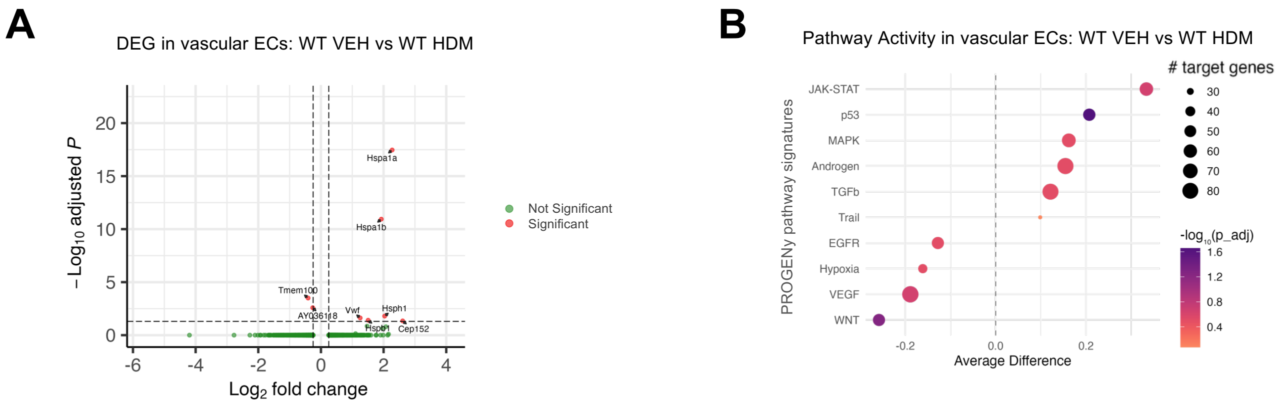

Supplementary Figure 11. TF and pathway enrichment analysis for Vascular ECs. A) Volcano plot showing downregulated and upregulated DEGs in vascular ECs from WT VEH and WT HDM mice. **B)** Pathway activity in vascular ECs from WT VEH and WT HDM mice. Circle size indicates the proportion of genes associated with each term that are represented in the dataset, while color denotes statistical significance based on the adjusted p-value.

				at 2 mg/mL total protein	
HDM lot #	Total protein (mg/vial)	Der p1 (μg/vial)	LPS (EU/vial)	Der p1 (μg/mL)	LPS (EU/mL)
378908	47.23	1989.04	192250	84.21	8139.29
410262	45.30	1832.50	5200000	80.90	229580.57

Supplementary Table 1. Composition of HDM extracts used in this study.

	Forward primer (5'–>3')	Reverse primer (5'–>3')
Il4	AGATGGATGTGCCAAACGTCC	AATATGCGAAGCACCTTGGAA
Il13	AGGAGCTTATTGAGGAGCTGA	TGGAGATGTTGGTCAGGGAAT
Il17a	CTTTCCCTCCGCATTGACAC	TTTAACTCCCTTGGCGCAAAA
Tnf	ACTTCGGGGTGATCGGTCCC	GCTACGACGTGGGCTACAGGCT
Rplp0 (36B4)	AGGCGTCCTCGTTGGAGTG	AGAGCTGGGTTGTTCTCCAG

Supplementary Table 2. Oligonucleotides used for RT-qPCR analysis.

Condition	Samples	Total Reads	Total Cells	Fraction Reads	Mean Reads/Cell	Median Genes/Cell	Median UMI/Cell	Total Genes	Filtered Cells
WT VEH	A1	188M	7,166	96.4%	26,248	1,014	1,938	21,382	5,671
	A2	199M	7,021	96.7%	28,364	1,249	2,640	21,756	5,538
WT HDM	B1	186M	7,490	97.0%	24,869	1,177	2,319	21,242	6,010
	B2	193M	7,918	91.2%	24,360	1,129	2,224	21,128	6,159
Il1b$^{-/-}$ VEH	C1	261M	7,119	96.2%	36,760	1,037	2,069	21,401	5,750
	C2	202M	7,863	96.7%	25,641	1,301	2,815	21,790	6,337
Il1b$^{-/-}$ HDM	D1	238M	12,024	97.3%	19,834	1,281	2,765	21,867	9,513
	D2	280M	8,423	92.5%	33,282	1,530	3,424	22,153	6,161

Supplementary Table 3. Quality control (QC) metrics for the scRNA-seq data. Table summarizing the sequencing and QC metrics across the four experimental conditions. “Total Cells” and other metrics were processed using Cell Ranger. “Filtered Cells” refers to the final cell counts after excluding technical artifacts such as doublets, ambient RNA, and dead cells, which were removed for downstream analysis.
